# Label matters: comparing gold nanoparticles and nanoshells with upconversion nanoparticles for quantitative lateral flow immunoassays

**DOI:** 10.1007/s00604-026-08015-5

**Published:** 2026-03-31

**Authors:** Eliška Macháčová, Jakub Máčala, Martin Kopecký, Saara Kuusinen, Tero Soukka, Zdeněk Farka

**Affiliations:** 1 Department of Biochemistry, Faculty of Science, Masaryk University, Kamenice 5, Brno, 625 00 Czech Republic; 2 Department of Life Technologies/Biotechnology, Faculty of Technology, University of Turku, Kiinamyllynkatu 10, Turku, 205 20 Finland

**Keywords:** Lateral flow immunoassay, Biomarker detection, Gold nanoparticle, Photon-upconversion nanoparticle, Human serum albumin, Prostate-specific antigen

## Abstract

Lateral flow immunoassay (LFIA) is a widely used point-of-care method for both qualitative and semi- or fully quantitative detection of clinical biomarkers. There has, however, been limited data available on direct comparison of different types of labels in the LFIA for quantitative analysis using the same non-competitive assay setup. In this study, two groups of antibody-conjugated nanoparticle labels are compared: conventional gold nanostructures with densitometry readout and upconversion nanoparticles (UCNPs) suitable for sensitive luminescence readout. First, the LFIA procedure was optimized using human serum albumin (HSA) as an analyte. In the group of gold-based labels, standard gold nanoparticles were compared with gold nanoshells (AuNSs). In the case of UCNP labels, doping with Er^3+^ and Tm^3+^ and surface modifications based on poly(ethylene glycol) and poly(acrylic acid) (PAA) were compared. The best representatives—AuNSs and PAA-Tm UCNPs—were successfully used to detect human serum albumin (HSA) in spiked urine samples. To assess assay versatility, prostate-specific antigen (PSA) was detected in serum. While AuNSs tended to aggregate in serum, the PAA-Tm UCNPs maintained their stability and achieved the limit of detection of 34 pg/mL. Finally, spiked serum samples were analyzed to evaluate detection trueness, achieving a strong correlation with the reference concentrations (recoveries ranging from 82 to 118%). We showed that both label choice and sample matrix are critical: while AuNSs and PAA-Tm UCNPs performed well in urine, PAA-Tm UCNPs excelled in the more complex serum matrix. Thus, PAA-Tm UCNPs were found to be the most suitable labels for biomarker quantitation, highlighting the strong potential of UCNPs for sensitive LFIA applications.

**Graphical abstract:**

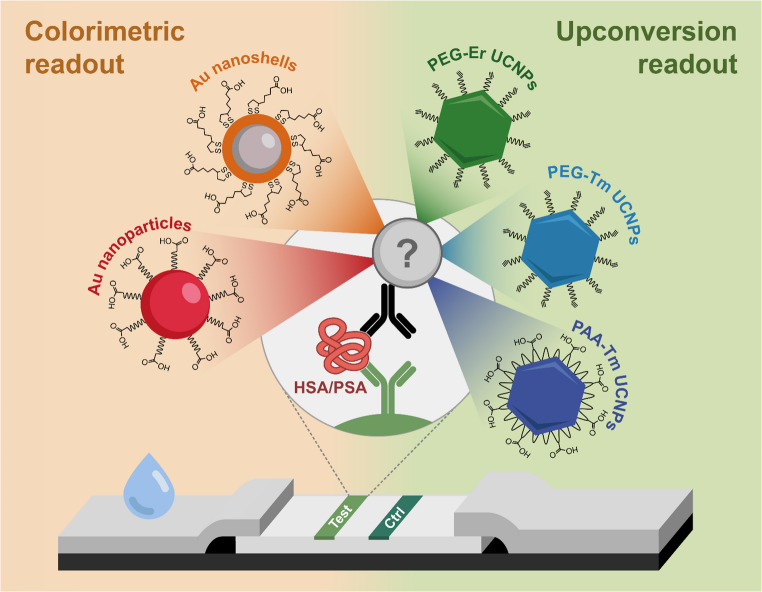

**Supplementary Information:**

The online version contains supplementary material available at 10.1007/s00604-026-08015-5.

## Introduction

With increasing demand for compact, simple, and rapid analytical methods, point-of-care (POC) testing has experienced significant growth. Unlike conventional laboratory testing, which requires sample pretreatment, transport, and bulky instrumentation, POC delivers results rapidly and directly at the site of need. A typical POC device comprises immobilized biorecognition components that interact with the target analyte in the sample, while the detection relies on biocatalytic or bioaffinity transduction combined with optical or electrochemical signal conversion [1, 2].

Lateral flow immunoassay (LFIA) is one of the most widely used and promising tools for POC testing. The LFIA strip consists of several membrane segments, with a nitrocellulose membrane serving as the primary component that includes test and control zones. The biorecognition element binds the analyte in the test zone, while part of the remaining unbound label is captured in the control zone, and the excess reagents migrate to the absorbent pad. The binding system typically exploits antibody-antigen interaction, but can also employ aptamer-analyte or DNA-DNA binding [3–5]. The key benefits of LFIA include reagent-free analysis, user-friendly operation, and the ability to provide rapid results, enabling immediate clinical decisions and improving patient prognosis. In addition, thanks to the low production costs, LFIA commercialized and expanded as a POC detection tool [4].

Conventional LFIAs are based on naked-eye readout of labels based on gold nanoparticles (AuNPs) accumulated in the test and control zones. This approach has the indisputable advantage of instrument-free signal evaluation, which is particularly helpful in applications such as home pregnancy testing. The ability of AuNPs to facilitate the visual detection of analytes complies with the World Health Organization ASSURED guidelines for the design of POC testing methods [6]. Consequently, LFIAs with AuNP-based labels have been adopted for the detection of a wide range of analytes [7]. However, in some applications, AuNP-based LFIA has shown limited sensitivity for detecting low-abundance biomarkers, the naked-eye readout provides only qualitative or semiquantitative results [8], and visual interpretation is prone to human errors [9]. Thus, for fully quantitative LFIAs, a reader-based detection is required, and for high-sensitivity assays, the colorimetric readout is often inadequate. Moreover, it was found that label surface modification is a key factor affecting LFIA performance [10], underscoring the need for further investigation in this direction. As an alternative, quantum dots (QDs) can be used in LFIAs with fluorescence readout. QDs are semiconductor nanocrystals with size-tunable optical properties, which are convenient for multiplexed LFIA analysis [11]. However, the use of QD-based labels is connected with several limitations, including their toxicity, the difficulty of surface modification, and autofluorescence of nitrocellulose exposed to visible excitation light [12]. Recently, photon-upconversion nanoparticles (UCNPs) have attracted attention as promising luminescent labels. UCNPs are typically composed of NaYF_4_ matrix doped with Yb^3+^ and Er^3+^ or Tm^3+^_,_ and are known for their anti-Stokes luminescence. This unique ability reduces the optical background by minimizing autofluorescence, improving detection sensitivity and enabling precise quantitation of the analyte. However, despite the advantages of UCNPs originating from their upconversion luminescence, non-specific interactions remain a crucial factor limiting the detection sensitivity [13]. Several types of surface modifications have been studied in LFIAs, with poly(ethylene glycol) (PEG) [14] and poly(acrylic acid) (PAA) [15] coatings being among the most common. UCNP-based LFIAs enabled the detection of different types of biomarkers, including cardiac troponin I [16], SARS-CoV-2 nucleoprotein [17], and prostate-specific antigen (PSA) [18]. However, previous studies have focused only on a single type of surface chemistry; therefore, a direct comparison of the performance of different coatings used in the single LFIA configuration is missing.

To address this issue, we compare the PEG- and PAA-modified UCNPs with different doping ions as labels in otherwise identical LFIA tests. Moreover, the performance of UCNP-based labels is compared with conventionally used LFIA labels, represented by AuNPs and gold nanoshells (AuNSs). A critical comparison of different labeling strategies will provide a foundation for further research in the LFIA field. Within the comparison, the labels were first optimized in LFIA for the quantitative detection of human serum albumin (HSA). HSA is the most abundant protein in blood plasma; however, it also has diagnostic value, as abnormal HSA levels in urine can be associated with diabetes, nephropathy, neurometabolic disorders, and other diseases [19]. Normal HSA concentration in the urine of healthy individuals does not exceed 30 µg/mL, while higher concentrations can indicate microalbuminuria [20]. Based on the obtained results, PAA-Tm UCNPs and AuNSs were selected as the most suitable labels for detecting HSA in spiked urine samples. Subsequently, to assess assay adaptability, the assay was modified for the determination of PSA in the spiked serum samples. PSA is the most common biomarker for prostate cancer, which is one of the most prevalent forms of cancer affecting men worldwide. In healthy men, the PSA level in the serum typically remains below 2.5 ng/mL, while concentrations above this threshold can indicate prostate cancer [21]. Importantly, detecting even lower PSA levels is essential, as radical prostatectomy typically reduces PSA concentration to nearly zero. Therefore, monitoring even slight changes in PSA at the pg/mL scale is critical for identifying potential cancer recurrence [22].

## Experimental section

### Chemicals and materials

Protein-free blocking buffer TBS (PF) and SuperBlock TBS (SB) were purchased from Thermo Fisher Scientific (USA). HSA (A8763), rabbit anti-HSA polyclonal antibody (A0433), fetal bovine serum (FBS), and Tween 20 were purchased from Merck (Germany). Mouse anti-HSA monoclonal antibody AL-01 was purchased from Exbio (Czech Republic). PSA (ab283430) and mouse anti-PSA monoclonal antibody (ab403) were purchased from Abcam (UK). Goat anti-PSA polyclonal antibody (AF1344) was purchased from R&D Systems (USA). Goat anti-mouse polyclonal antibody (115-005-003) was purchased from Jackson ImmunoResearch (UK). Carboxyl-coated 40-nm gold nanospheres (PEG_12_-carboxylic acid surface) and 150-nm gold nanoshells (lipoic acid surface) were purchased from nanoComposix (USA). All other chemicals were purchased from Penta (Czech Republic), P-LAB (Czech Republic), or Carl Roth (Germany).

The membrane components used for the lateral flow strips included an FF120HP Plus Thick nitrocellulose membrane, a CF3 sample pad cellulose membrane, a CF5 absorbent pad cellulose membrane, and an ST17 conjugate pad glass fiber membrane, all purchased from Cytiva (USA). Additionally, Hi-Flow Plus HF12004 and HF075 nitrocellulose membranes were purchased from Merck (Germany).

The coating buffers included acetate buffer (5 mM acetic acid, 1% methanol; pH 4.4), Tris-buffered saline (50 mM Tris, 150 mM NaCl, 0.05% NaN_3_, 1% methanol; pH 7.5), carbonate buffer (50 mM NaHCO_3_/Na_2_CO_3_, 0.05% NaN_3_; pH 9.6), phosphate buffer (50 mM NaH_2_PO_4_/Na_2_HPO_4_; pH 7.4), and Tris buffer (10 mM Tris, 5% ethanol, 1% sucrose; pH 8). Furthermore, assay buffer (50 mM Tris, 150 mM NaCl, 1 mM KF, 0.1% PEG (6 kDa), 0.02% Tween 20, 0.05% NaN_3_, 10% SB; pH 7.5) and washing buffer (50 mM Tris, 150 mM NaCl, 0.05% NaN_3_, 0.05% Tween 20; pH 7.5) were used. The blocking buffers had the same composition as the washing buffer, but different blocking agents were introduced: 50% SB, 50% PF, 10% FBS, and mixtures of 20% SB and 10% FBS, 20% PF and 10% FBS, and 20% SB and 20% PF.

Protocols for the synthesis of NaYF_4_:Yb^3+^,Er^3+^ (58 nm) and NaYF_4_:Yb^3+^,Tm^3+^ (60 or 30 nm) UCNPs, their antibody conjugation, characterization with transmission electron microscopy (TEM) and dynamic light scattering (DLS), and emission spectra measurements, as well as the antibody conjugation of AuNPs and AuNSs with subsequent TEM and DLS characterization, protocols for experiments verifying the successful conjugation of nanoparticles with antibodies by absorption spectra measurements (Au-based labels) and upconversion-linked immunosorbent assay (UCNP-based labels), and reference enzyme-linked immunosorbent assay (ELISA) experiments, are provided in the Supplementary Information.

### LFIA strip preparation

Three different types of LFIA strips were used throughout the experiments: strips with a conjugate pad and pipette-based spot deposition of antibodies, dip-stick tests (i.e., strips without a conjugate pad) with pipette-based spot deposition of antibodies, and dip-stick tests with piezo-driven line deposition of antibodies.

**Fig. 1:**
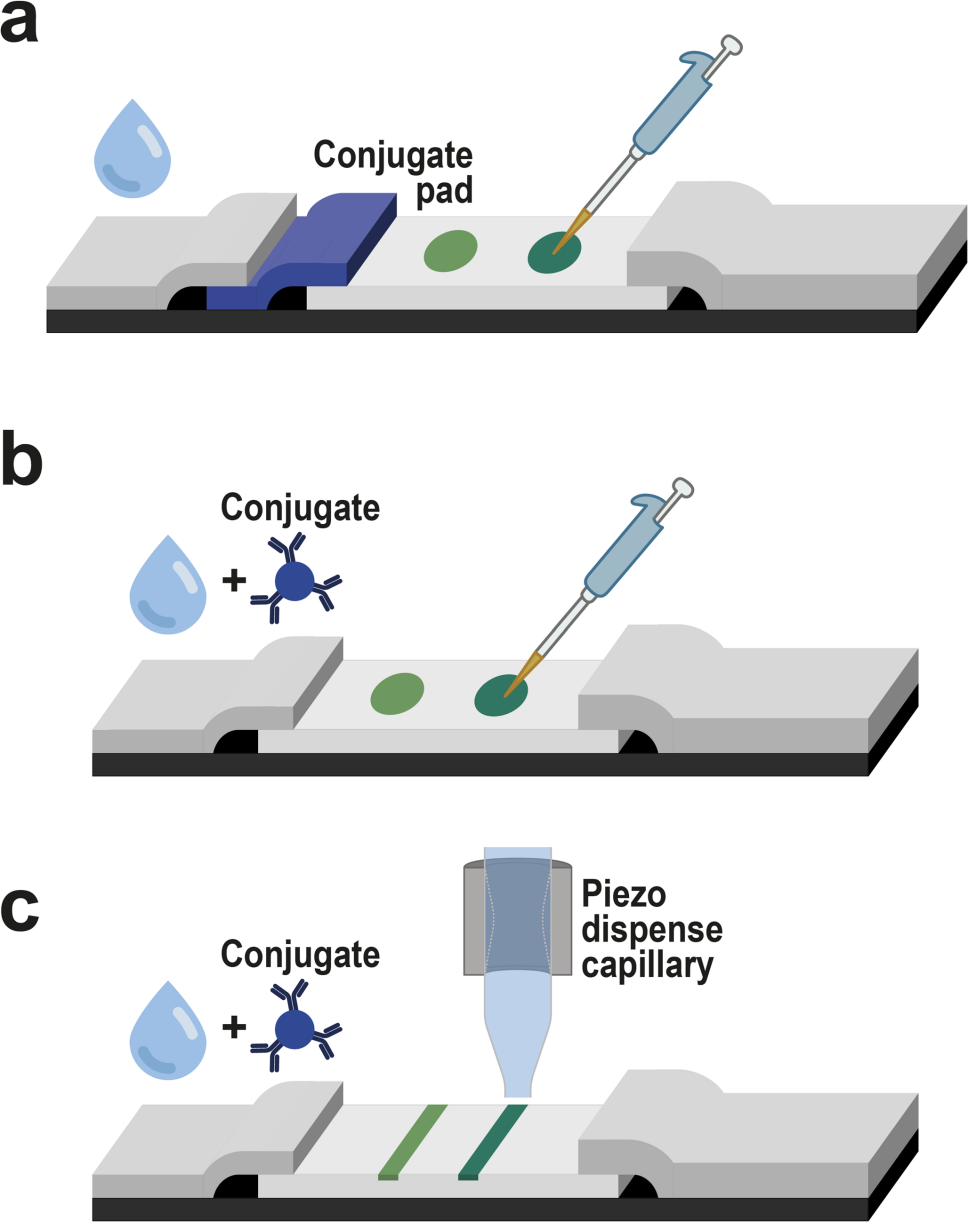
Schematic illustration of three types of LFIA strips used in this study: **a** LFIA strip with conjugate pad and pipette-based antibody spot deposition, **b** dip-stick format (without conjugate pad) with pipette-based antibody spot deposition, and **c** dip-stick format with piezo-driven antibody line deposition

For strips with a conjugate pad and pipette-based spot deposition (Fig. 1a), a nitrocellulose membrane (2.5 cm wide; length determined by the number of strips, considering 0.5 cm per strip) was affixed to a plastic support using double-sided adhesive tape. A cellulose absorbent pad (3 cm wide) was then attached to the support along the entire length of the nitrocellulose membrane, ensuring a 1 mm overlap. At this stage, the membranes were cut into 0.5-cm strips. Circular control and test zones were prepared by depositing 1 µL of polyclonal anti-mouse antibody (0.4 mg/mL) in the control zone and 1 µL of either polyclonal anti-HSA or polyclonal anti-PSA antibody (0.5 mg/mL) in the test zone, resulting in 400 and 500 ng of antibody in the control and the test zone, respectively. Acetate buffer was used to dilute the antibodies. After establishing the control and the test zones, the strips were dried at room temperature for 15 min. To block the remaining nitrocellulose binding sites, a solution of 20% PF and 10% FBS in the washing buffer was applied using a spray dispenser, followed by 1 h of drying at 37 °C. The sample pads (1 × 0.5 cm) were blocked in the same manner. The conjugate pad, made of a glass fiber membrane (0.5 × 0.5 cm), was soaked with 10 µL of nanoparticles conjugated with either monoclonal anti-HSA or monoclonal anti-PSA antibody and dried for 30 min at 37 °C. Undiluted gold nanostructure conjugate dispersions with an optical density of 20 were used in colorimetric LFIA. For upconversion-based LFIA, the UCNP conjugate dispersion was diluted to 6.5 µg/mL in the assay buffer. The dried conjugate and sample pads were subsequently attached to strips with a 1 mm overlap.

For dip-stick tests with pipette-based spot deposition (Fig. 1b), the nitrocellulose membrane, absorbent pad, and sample pad (1 cm wide) were attached to the plastic support with 1 mm overlaps, and the membranes were cut into 0.5 cm strips. The strips were coated with antibodies and blocked as described above.

For dip-stick tests with piezo-driven line deposition (Fig. 1c), antibodies were dispensed onto the test and control lines on the nitrocellulose membrane using the sciFLEXARRAYER S3 (Scienion, Germany), and the membrane was then attached to a plastic support along with an absorbent and a sample pad. The dispenser settings were adjusted to a spot distance of 100 μm to produce a coherent line across the 5-mm wide strip (50 spots overall). Each spot consisted of 50 drops of ~ 350 pL (concentration of 0.4 and 0.5 mg/mL for anti-mouse and anti-HSA or anti-PSA antibody, respectively). A piezo dispense capillary with a type 4 coating (Scienion, Germany) was used, with the voltage, pulse width, and frequency set to 80 V, 50 µs, and 500 Hz, respectively. This resulted in 350 and 437.5 ng of antibody in the control and the test line, respectively.

### LFIA assay procedure

The assembled strips with conjugate pad were dipped into the wells of a non-binding microtiter plate (Greiner Bio-One, Austria) containing 50 µL of HSA standard diluted in the assay buffer. Once the liquid was fully aspirated, the strips were dipped into 100 µL of washing buffer in microtiter plate wells until the entire volume was absorbed, followed by drying at 37 °C for 1 h. In the dip-stick approach, 50 µL of HSA or 40 µL of PSA standard diluted in assay buffer was mixed with 10 µL of the conjugate solution (concentration of 6.5 µg/mL for UCNPs and optical density of 20 for Au nanostructures), and the mixture was incubated for 1 h at 300 rpm on a Titramax 101 shaker (Heidolph, Germany). Dip-stick tests were subsequently immersed in the mixture, washed, and dried as described above.

For HSA detection in real samples, urine collected from two healthy volunteers was purified using Amicon Ultra centrifugal filters with a molecular weight cut-off of 10 kDa (Merck, Germany) and stored at − 20 °C. Before the assay, the urine was diluted to 50% with assay buffer and used to prepare HSA calibration standards.

For PSA detection in real samples, blood for the serum pool was collected from three healthy female volunteers with informed consent, in compliance with the Declaration of Helsinki. No individual samples were analyzed; the blood was used solely as a blank matrix to be spiked with PSA. Blood was collected into a Vacuette serum tube (Greiner Bio-One, Austria) and processed according to the manufacturer’s instructions. After pooling, the serum was stored at − 20 °C. Additionally, female and male human plasma samples treated with 4% sodium citrate were bought from AGEL (Czech Republic) and stored at − 20 °C. Before use, both serum and plasma were further centrifuged (30 min at 3,300 × *g*), and the upper liquid was filtered through a 0.45-µm syringe filter to remove the denatured protein mass. Subsequently, the serum or plasma was diluted to 25% with the assay buffer and either used to prepare standards or spiked with known amounts of PSA.

### Data acquisition and evaluation

Colorimetric LFIA strips were captured using a Fusion FX 6 Edge imaging system (Vilber, France) equipped with a CCD camera, and the acquired 16-bit grayscale images were inverted using ImageJ software (National Institutes of Health, USA). For upconversion-based LFIA, the strips were scanned using an Upcon S-Pro reader (Labrox, Finland) utilizing a 980-nm laser and a 976/60 nm excitation filter. The detection of Er-doped labels employed a D800 dichroic mirror and a 540/60 nm emission filter, and the Tm-doped labels were detected using a D900 dichroic mirror and an 810/40 nm emission filter. The scanning was performed in a two-dimensional mode, with the step size of 0.5 mm for pipette-based deposition and 0.25 mm for the piezo-printed strips. The integration time, emission spot size, and relative laser power were adjusted to 500 ms, 4 mm, and 10%, respectively. Data from the upconversion-based LFIAs were imported into ImageJ, and 32-bit pseudocolor images were created.

The average test zone intensities were calculated using ImageJ and further analyzed in OriginPro 2023 (OriginLab, USA). For pipette-based antibody spot deposition, intensity was measured from the circular test zone area, whereas for piezo-driven antibody line deposition, it was evaluated as the integrated peak maximum. In this approach, pixel intensities in the test zone were averaged in the horizontal direction, and an intensity profile was plotted. Subsequently, the maximum value of the plot was used as the test zone signal intensity [4]. In the optimization experiments, the average signal intensity in the test zone was divided by the average signal intensity in the nitrocellulose background of the strips to determine the signal-to-background (*S*/*B*) ratio. The final values were averaged for replicates and are presented in column graphs. In the experiments to construct a calibration curve, the test zone intensities were averaged for replicates and fitted using a four-parameter logistic function:

$$\:y=\frac{{A}_{1}-{A}_{2}}{1+{\left(\frac{x}{{x}_{0}}\right)}^{p}}+{A}_{2}$$

where *A*_1_ represents the curve minimum, *A*_2_ the curve maximum, *x*_0_ the inflection point, and *p* the slope of the curve at the inflection point.

The LODs were estimated from the calibration curve as the analyte concentrations corresponding to the *y*_LOD_ value:

$$\:{y}_{\mathrm{L}\mathrm{O}\mathrm{D}}={A}_{1}+3{s}_{B}$$

where *A*_1_ is the curve minimum from the logistic fit and *s*_B_ is the standard deviation of the blank.

The working ranges were evaluated from the fits as the intervals between the analyte concentrations required to obtain 20% (EC_20_) and 80% (EC_80_) of the maximum signal.

## Results and discussion

### Optimization of the LFIA procedure for HSA detection

Determining optimal conditions is essential for developing an immunoassay with the best possible performance. For initial optimizations, the PEG-coated Er-doped UCNPs were selected as a label based on our previous study describing the development of a nitrocellulose-based dot-blot immunoassay [12]. HSA (at a concentration of 10 or 100 ng/mL) was used as a model analyte to thoroughly optimize assay parameters and achieve the highest possible performance. Therefore, the optimizations aimed to achieve high sensitivity of HSA detection, although this is not directly relevant to the HSA cut-off concentration for microalbuminuria. Antibodies were deposited in the test and the control zone as circular spots by pipetting 1 µL of the respective solution onto a nitrocellulose membrane. Most optimization experiments did not aim to determine the LOD, but rather the *S*/*B* ratio, as a high *S*/*B* is a key prerequisite for achieving low LODs. Characterization of all labels using TEM and DLS is presented in Fig. S1, S2, S3, and Table S1. The functionalization of particle surfaces with anti-analyte antibodies was confirmed by absorption spectra measurements for conjugated gold nanostructures (Fig. S4) and by an upconversion-linked immunosorbent assay for UCNP conjugates (Fig. S5). Emission spectra of UCNPs with different doping ions are provided in Fig. S6. Additionally, the stability of the conjugates was examined using DLS (Fig. S7). Only minor changes in particle size and PdI were observed within the 5-week test interval, confirming that the particles can be used even after long-term storage.

**Fig. 2:**
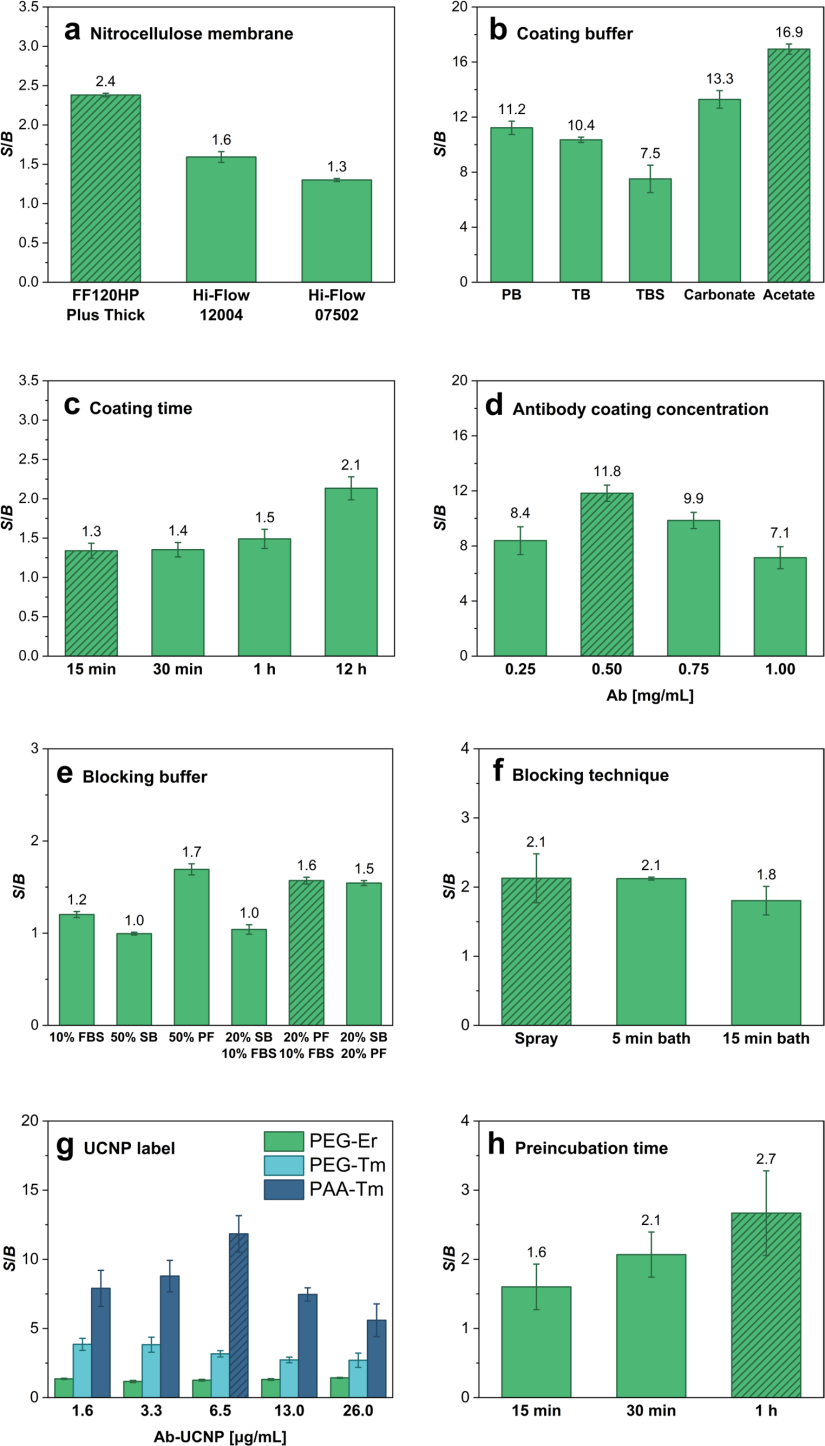
Optimization of the LFIA assay with pipette-based spot deposition of antibodies, PEG-Er UCNPs as a label, and HSA as an analyte. Optimization of **a** nitrocellulose membrane, **b** coating buffer, **c** coating time, **d** antibody coating concentration, **e** blocking buffer, **f** blocking technique, **g** type and dilution of UCNP-based label, and **h** preincubation time of the dip-stick method. The concentration of HSA was 10 ng/mL (panels **a**, **c**, **e**, **f**, **g**, and **h**) or 100 ng/mL (panels **b** and **d**). Error bars represent standard deviations, and hatched columns indicate the optimal conditions

The first step of LFIA optimization was to select the optimal nitrocellulose membrane. A suitable membrane should exhibit sufficient binding capacity to capture antibodies while allowing efficient capillary flow of the sample toward the absorbent pad. Three different membranes were compared (Fig. 2a). The FF120 HP Plus Thick membrane provided efficient capillary flow and achieved the highest *S*/*B* ratio of 2.4; therefore, it was selected for subsequent experiments. Next, the coating buffer used to dilute the antibody was optimized (Fig. 2b). An optimal coating buffer should provide a highly stable solution of diluted antibody and enable effective antibody binding to nitrocellulose. All tested buffers contained 1% methanol, which is known to improve the immobilization on the membrane surface [23]. Among the tested options, the highest *S*/*B* ratio was obtained with acetate buffer, which was thus selected for further experiments. Subsequently, it was necessary to determine the optimal coating time providing sufficient antibody binding while keeping the preparation time to a minimum (Fig. 2c). Although the *S*/*B* ratio gradually increased with the increasing coating time, this difference was insignificant (increasing the coating time 48 times from 15 min to 12 h resulted in only a 1.6-fold increase in *S/B*). Therefore, considering the time requirements, 15 min was selected as optimal. Subsequently, the antibody coating concentration was optimized (Fig. 2d). Determining a suitable concentration is crucial for assay performance, as insufficient antibody concentration can result in low capture of the analyte-label complexes, while an excessive concentration can lead to increased non-specific binding in the test zone. The highest *S*/*B* ratio of 11.8 was achieved at a concentration of 0.5 µg/mL; therefore, this concentration was used for further experiments.

Next, different blocking strategies were tested (Fig. 2e). If the unoccupied binding sites on nitrocellulose are not efficiently blocked, non-specific adsorption on the porous membrane surface can occur, leading to a high assay background and deterioration of the LOD. Three blocking agents and their combinations were compared: FBS, SB, and PF. The results indicated similar good performance for several options, all of which included some portion of PF. However, a blocking solution containing 20% PF and 10% FBS was chosen for subsequent use because it retained sufficiently strong signals in the test and control zones while providing a low background in the areas of nitrocellulose outside the zones. Additionally, three blocking approaches were tested (Fig. 2f). The blocking solution was applied either by spraying it onto the surface of the nitrocellulose or by submerging the entire strip in the bath for 5 or15 min. Although bath blocking resulted in a lower background, it may also have washed out the deposited antibodies, leading to reduced test zone signals. Thus, although the *S*/*B* values of the spray blocking and 5-min bath were similar, the spray approach was selected because it reduced the risk of washing the antibodies out of the nitrocellulose.

In the label optimization, various concentrations of UCNPs with two different types of surface modification were compared (Fig. 2g). The first surface modification utilized the binding of the neridronate-PEG-alkyne linker to the surface of either Er-doped (PEG-Er) or Tm-doped (PEG-Tm) UCNPs. Neridronate binds to the UCNP through coordination between lanthanide ions on the nanoparticle surface and the two phosphonate groups of neridronate, PEG stabilizes nanoparticle dispersion and prevents non-specific adsorption, and alkyne is employed in the click reaction that yields a covalent bond with antibodies. The second approach involved coating Tm-doped UCNPs with poly(acrylic acid) (PAA-Tm). PAA binds to the UCNPs through coordination bonds between the lanthanide atoms on the nanoparticle surface and the carboxyl groups of PAA. The carboxyl groups further enable conjugation of PAA-coated UCNPs with the amine groups of antibodies *via* EDC/sulfo-NHS chemistry. The tested UCNP concentrations ranged from 1.6 to 26 µg/mL. Among the PEG-modified conjugates, the Tm-doped particles exhibited approximately 2–3 times higher signals across the entire tested concentration range compared to the Er-doped particles. However, the comparison of Tm-doped conjugates with different surface modifications revealed that the PAA-coated Tm-doped UCNPs showed an even higher signal intensity across the tested concentration range, with a concentration of 6.5 µg/mL achieving the highest *S*/*B* of 12.

There are two common methods to perform LFIA. The first one uses a glass fiber membrane as a conjugate pad with a dried label conjugate dispensed before the immunoassay is carried out. The advantage of this approach is the generally shorter assay duration, as all the test components are present on the strip and only the sample is added to the sample pad. The second option, based on the dip-stick format, involves preincubating the sample with the labels, followed by submerging the sample pad of the assembled test strip (without a conjugate pad) in this mixture. The limitation of this approach is the longer total assay duration due to the preincubation step. However, analyte molecules have more time to bind to the labels during the preincubation, which can result in a higher sensitivity. Moreover, the diffusion in the mixture is more pronounced compared to the use of a conjugate pad, further enhancing binding efficiency. Before comparing the two approaches, it was necessary to optimize the preincubation time (Fig. 2h). Incubation times of 15–60 min were tested, and the highest *S*/*B* ratio of 2.7 was achieved for a 60-min preincubation. Although a longer preincubation might yield an even higher *S*/*B* ratio, this option was not considered to keep the total assay duration reasonable.

### Comparison of Au nanostructures and UCNPs as labels in LFIA

**Fig. 3:**
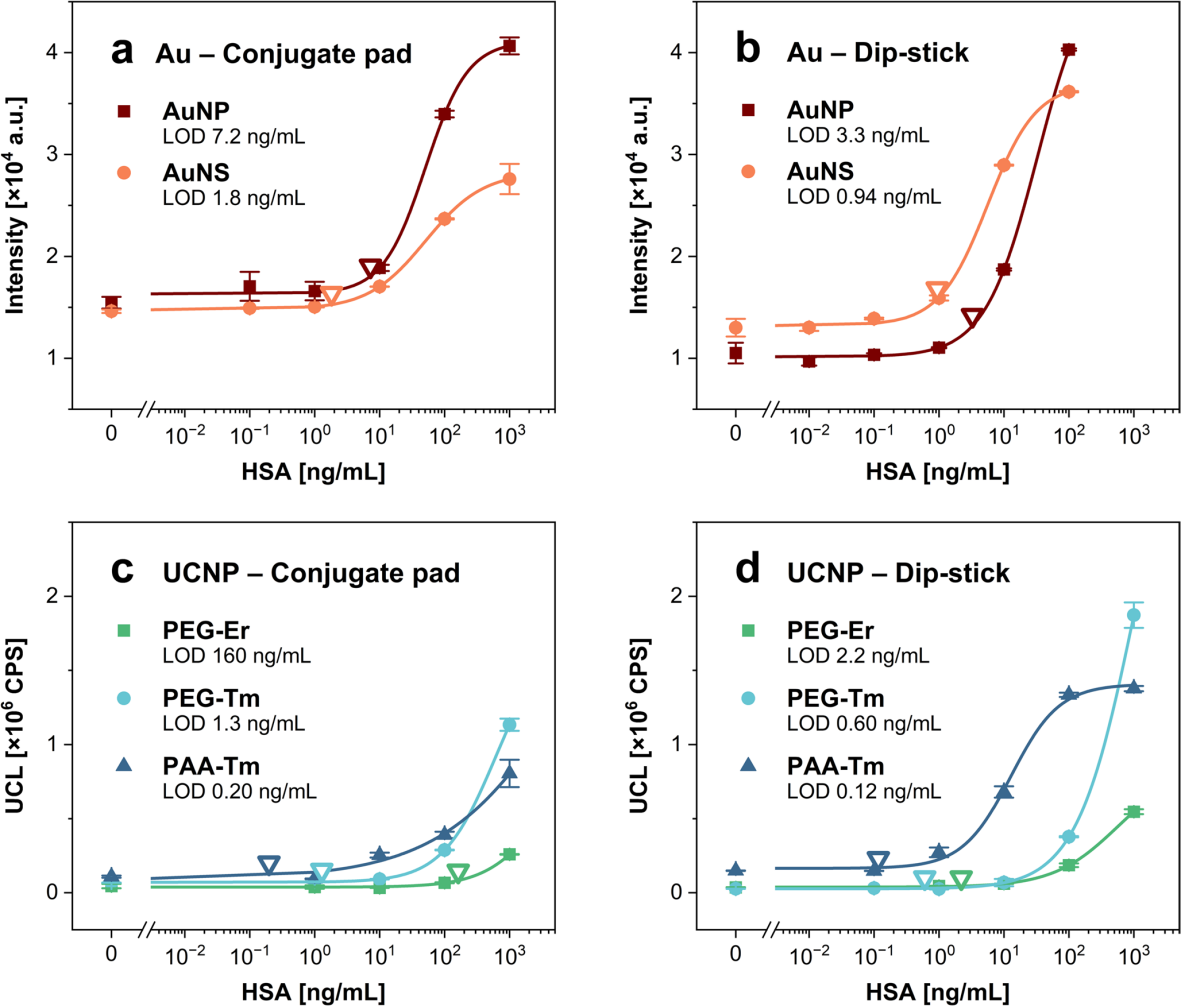
Comparison of LFIA approaches for HSA detection using the conjugate pad and dip-stick method. Calibration curves obtained using Au-based nanostructure labels in **a** the conjugate pad and **b** the dip-stick format configuration, and the results with UCNP-based labels in **c** the conjugate pad and **d** the dip-stick format. Error bars represent standard deviations, and empty triangles indicate the LODs

With the optimized conditions, the assay approach involving a conjugate pad was compared with the dip-stick method, while also incorporating colorimetric labels—specifically red 40-nm AuNPs and green 150-nm AuNSs. For these experiments, whole calibration curves were constructed, and the LODs were calculated. The LODs for the gold-based AuNP and AuNS labels were 7.2 and 1.8 ng/mL, respectively, using the strip with a conjugate pad (Fig. 3a), and 3.3 and 0.94 ng/mL with the dip-stick method (Fig. 3b). The LODs achieved with the UCNP-based labels were 160 ng/mL (strip with a conjugate pad; Fig. 3c) and 2.2 ng/mL (dip-stick; Fig. 3d) for PEG-Er, 1.3 and 0.6 ng/mL for PEG-Tm, and 0.2 and 0.12 ng/mL for PAA-Tm. The analytical parameters of the respective calibration curves are summarized in Table S2. The results indicated that among the tested gold-based nanostructures, AuNSs provided the lowest LODs, while PAA-Tm showed to be the most suitable UCNP-based label. Therefore, these two label types were selected for further experiments, as the ability to detect low analyte concentrations is essential for the development of sensitive quantitative LFIAs. The difference between Tm-doped and Er-doped UCNPs may be partially attributed to variations in the optical detection setup, since different dichroic mirrors and emission filters are used for the respective emissions of Er- or Tm-doped UCNPs. Compared to AuNSs, the use of PAA-Tm UCNPs in LFIA utilizing the preincubation step yielded an 8-fold lower LOD. The obtained results showed that the dip-stick approach provides lower LODs for all the tested labels. This improved assay performance was probably connected with the solution mixing during preincubation. As a result, the diffusion of assay components was enhanced, and the time available for the analyte molecules to bind to the antibodies on the label surface was increased, further supporting efficient antibody-antigen binding.

### Enhancement of the LFIA performance with piezo-driven antibody deposition

The deposition of antibodies on nitrocellulose using a piezo-driven low-volume dispensing system was investigated for its potential to improve the assay performance. Automated low-volume dispensing enables the concentration of antibodies into a narrow line, subsequently allowing for the spatial concentration of labels in a smaller area. The system was set up to deposit the antibody solution by dispensing droplets onto the nitrocellulose surface, forming a coherent test or control line. Otherwise, the LFIA procedure was identical to that used with the pipette deposition. The performance of the LFIA strips utilizing the piezo-driven antibody line deposition was first tested for HSA detection in the assay buffer. The best results were again obtained using AuNS and PAA-Tm UCNP labels. In the case of AuNSs, an LOD of 76 pg/mL was achieved by utilizing the piezo-driven dispensing (Fig. S8a and 4a), which was 12-fold better than that obtained using pipette deposition. In comparison, the PAA-Tm UCNPs achieved an LOD of 32 pg/mL (Fig. S8b and 4b), representing a 3.8-fold improvement over manual antibody deposition. Moreover, the LOD obtained with PAA-Tm UCNPs was 2.4 times lower compared to AuNSs, highlighting the superior sensitivity of UCNP-based LFIA. The analytical parameters of the respective calibration curves are summarized in Table S3. The data obtained with both label types showed the advantages of using the piezo-driven dispenser for antibody deposition, as assays using this approach achieved significantly lower LODs than pipette dispensing. The piezo-driven dispensing system ensured even antibody deposition across the entire strip width, allowing all analyte-label complexes to be carried with the liquid flow across the test zone. In contrast, when antibodies were deposited with a pipette in the center of the strip, a portion of the analyte-label complexes moved around the test zone, reducing the obtained signal. Furthermore, the automated deposition ensured that the results were consistent and reproducible.

**Fig. 4:**
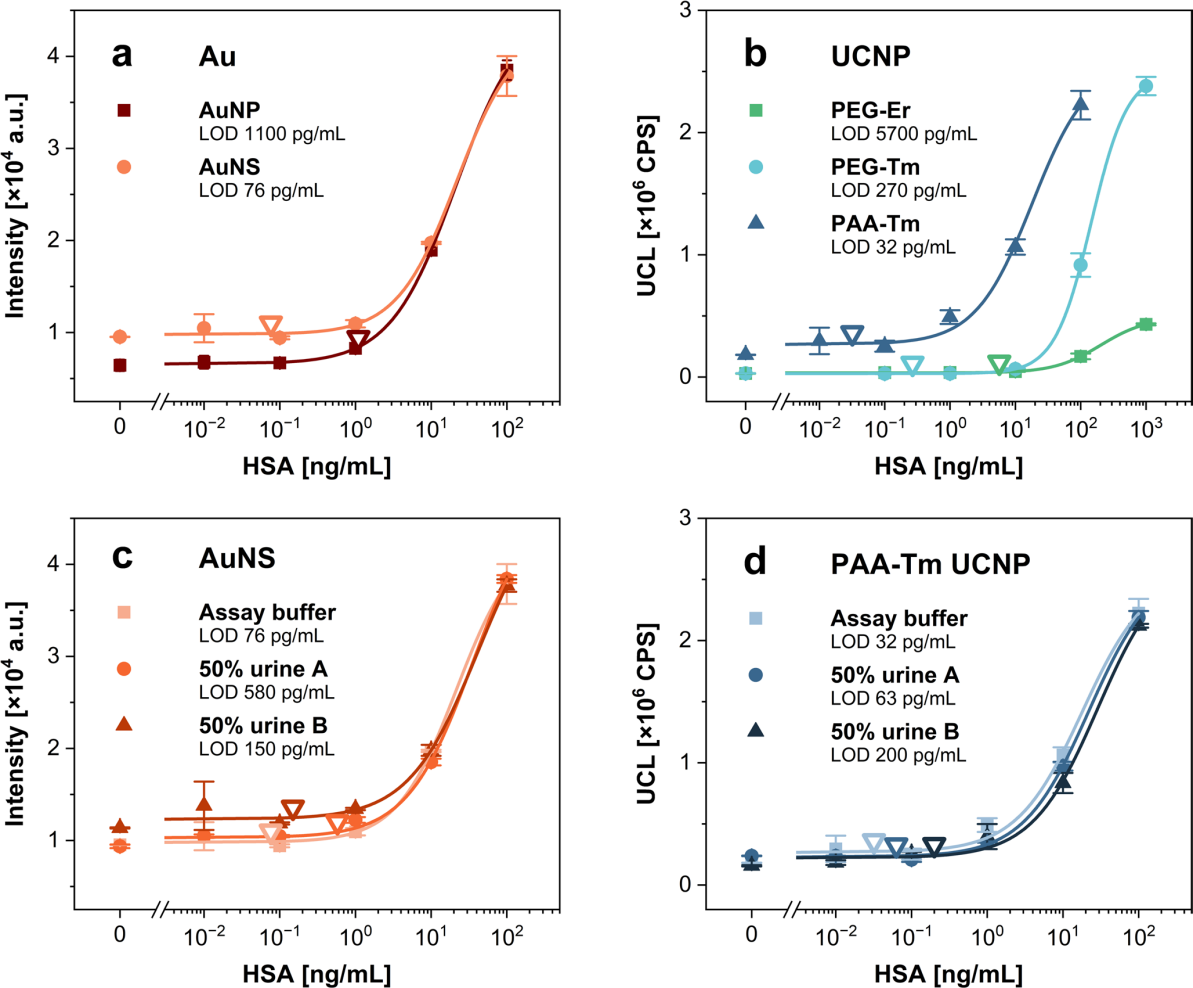
Comparison of different labels in LFIA based on piezo-driven antibody deposition. Calibration curves for HSA detection in the assay buffer utilizing **a** Au-based nanostructures and **b** UCNPs. Determination of HSA in urine samples utilizing **c** AuNS and **d** PAA-Tm UCNP labels; calibration curves in the assay buffer are included for reference. Error bars represent standard deviations, and empty triangles indicate the LODs

The immunoassay performance is typically negatively affected by the complex sample matrix, due to the possible presence of various components that can lead to an increased level of non-specific interactions. Therefore, to test the performance of the developed LFIA in complex matrices, the assay was performed in spiked urine samples from two healthy volunteers. The samples are typically diluted to minimize the potential adverse effects of the sample matrix, which can limit the assay performance. Moreover, the dilution helps reduce biological variability within different patient samples [12]. Because urine composition (e.g., pH, ionic strength, and protein concentration) can vary significantly depending on hydration status, the urine was diluted to 50% with the assay buffer. Furthermore, to mitigate the risk that the results of clinical analysis of unknown samples fall outside the assay working range, several urine dilutions can be prepared and analyzed. This way, the dilution that provides an HSA concentration within the working range of the assay can be selected, and the original HSA concentration in the sample can be determined by multiplying the concentration found in the diluted sample by the respective dilution factor. LFIA with AuNS labels achieved LODs of 580 and 150 pg/mL for samples A and B, respectively (Fig. 4c). The significant difference between the LODs determined in the two urine samples while utilizing the AuNSs might be caused by the slight aggregation of labels, which was studied in more depth for the PSA detection. The assay utilizing the PAA-Tm UCNP labels resulted in LODs of 63 and 200 pg/mL for samples A and B, respectively (Fig. 4d). This represents only a slight deterioration compared to the assay in the buffer (approximately 2× and 6× higher LODs for urine samples A and B, respectively), highlighting the exceptional usability of UCNPs for the analysis of complex biological samples.

### LFIA for PSA detection

**Fig. 5:**
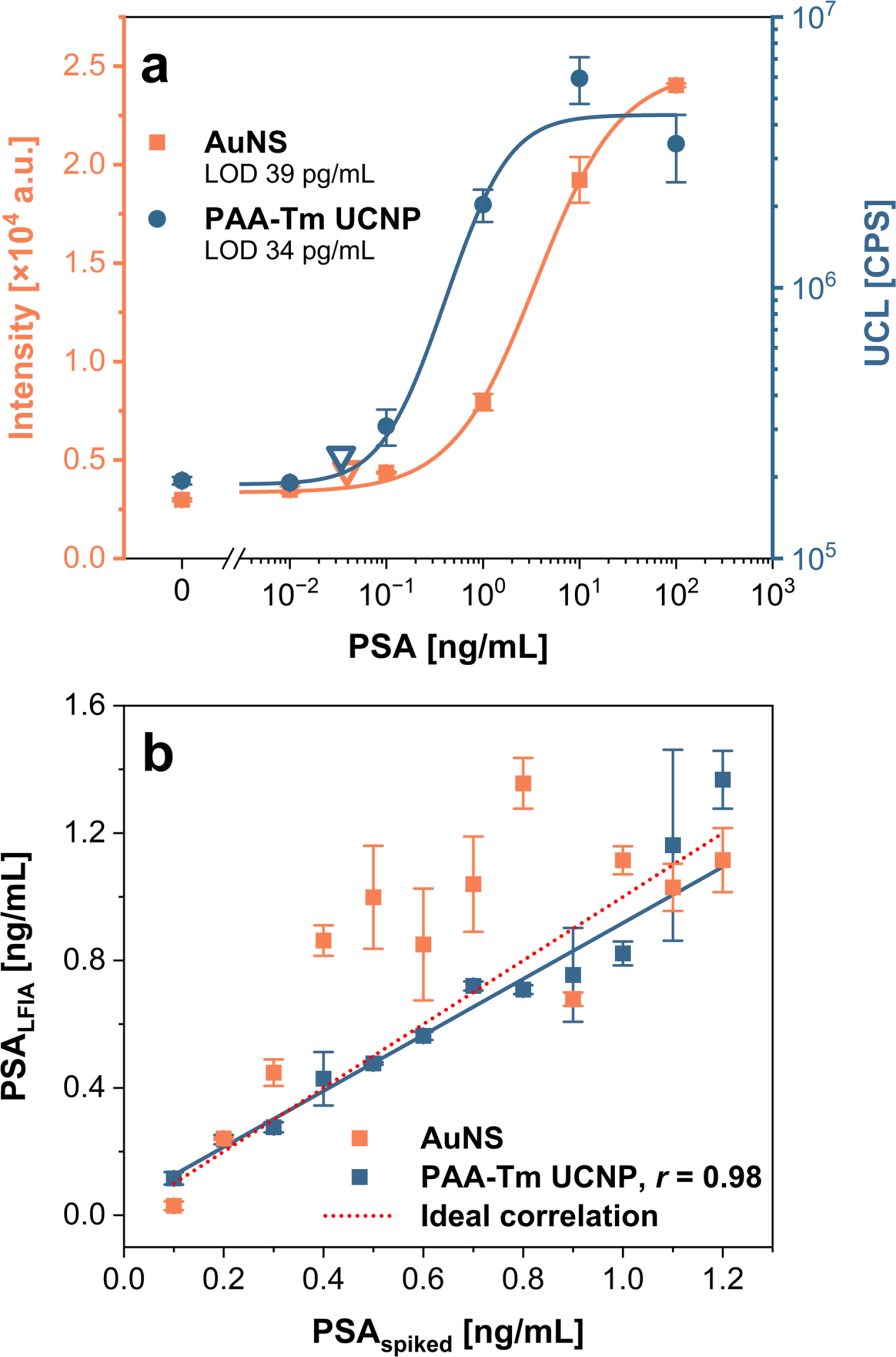
Determination of PSA in serum samples using LFIA with AuNS and PAA-Tm UCNP labels. **a** Calibration curve and **b** correlation between the spiked concentrations and the LFIA results. Error bars represent standard deviations, and empty triangles indicate the LODs

To demonstrate the adaptability of the developed assay for other analytes, PSA—the key prostate cancer biomarker—was detected using the optimized procedure. The analysis was carried out in spiked 25% serum, the sample matrix in which PSA is routinely tested in clinical laboratories [24]. The particular dilution factor was based on our previous work [13, 25]. Serum was obtained from female volunteers to minimize the possible PSA levels naturally present in the sample. First, detection was carried out using AuNS labels. However, after a 1-hour preincubation, visually observable aggregates were formed in all wells. The aggregation of AuNSs in the serum was confirmed by DLS analysis (Fig. S9a), which showed broad peaks at larger sizes (peak maximum at approximately 1 μm) corresponding to aggregates. The dispersion in the wells was thoroughly mixed until all visually observable aggregates disappeared before the test strip was immersed. It was possible to obtain the calibration curve (Fig. 5a), showing an LOD of 39 pg/mL (156 pg/mL considering the serum dilution); however, the use of aggregation-prone labels is not suitable for reliable analysis of real samples, as aggregation may compromise the accuracy and reproducibility of the assay.

Subsequently, detection was performed using PAA-Tm UCNPs. To determine whether PAA-Tm UCNPs were also subject to aggregation in the serum matrix, DLS measurements were performed (Fig. S9b). A slight broadening of the peak was observed, indicating some aggregate formation, but to a much lesser extent than in the case of AuNS labels. The increased stability of UCNPs was likely due to the PAA coating, which stabilized the particle dispersion. The assay using UCNPs (Fig. 5a) achieved an LOD of 34 pg/mL (136 pg/mL considering the serum dilution), similar to that obtained with AuNSs. Nevertheless, PAA-Tm UCNP labels were shown to be more suitable for analyzing serum samples due to their lower tendency to aggregate, providing more robust analysis.

To assess the trueness of the PSA determination, the serum samples were spiked with known concentrations of PSA and analyzed with the developed LFIA assays based on AuNS and PAA-Tm UCNP labels (Fig. 5b). As the concentration threshold of PSA in serum that indicates possible health problems is 2.5 ng/mL [21] and the detection was performed in 25% serum, the analyzed concentrations ranged from 0.1 to 1.2 ng/mL, corresponding to 0.4 to 4.8 ng/mL in the undiluted sample. The data obtained with AuNSs showed high deviations from the reference values (recovery rates ranging from 30 to 215%), which can be attributed to label aggregation negatively affecting assay trueness. In contrast, the use of the PAA-Tm UCNP labels yielded a strong correlation with reference levels, with recovery rates from 82 to 118%. These results highlight the advantages of PAA-Tm UCNPs over AuNSs for the sensitive and accurate determination of PSA in the complex serum matrix, clearly demonstrating the potential of UCNP-based LFIA for clinical sample analysis.

Furthermore, to demonstrate the versatility of LFIA with PAA-Tm UCNP labels across various biological matrices, plasma samples were analyzed. First, 25% female plasma—expected to contain no PSA—was spiked and used to construct a calibration curve for this matrix. Subsequently, LFIA detection of PSA was performed in a 25% male plasma sample. The found PSA concentration was below the LOD of the assay, which aligns with expectations, as the plasma was provided by a healthy donor. Therefore, a calibration curve was also constructed using the male plasma sample after spiking to further demonstrate that the biological variability of the samples does not significantly influence the assay performance. As shown in Fig. S10, the LODs in plasma (64 and 80 pg/mL for male and female sample, respectively) were only slightly higher than those obtained in serum (34 pg/mL), thereby confirming the versatility of the developed LFIA. In addition, to verify long-term storage stability, calibration in the same female plasma was performed after 1 month of storage of both LFIA strips and the PAA-Tm UCNP conjugate. No significant differences were observed among the calibration curves, and the assay achieved an LOD of 84 pg/mL, which agrees with the LOD of 80 pg/mL obtained with fresh components. This result thus confirms the high storage stability of all assay components.

To evaluate the trueness of the LFIA assay, female plasma was spiked with varying PSA concentrations and tested using both LFIA and the standard ELISA. For this purpose, an ELISA calibration curve was constructed in 25% plasma, achieving an LOD of 1.2 pg/mL (Fig. S11). Both methods enabled reliable detection of the target analyte, with recoveries ranging from 97 to 116% (Table S4). This confirms the strong correlation between LFIA and ELISA results and demonstrates that the developed LFIA is suitable for determining PSA with high trueness. In addition, the spiking experiments included concentrations above 10 ng/mL, successfully demonstrating that sample dilution can be used to overcome the hook effect observed in the case of high PSA concentrations.

### Comparison of different AuNP- and UCNP-based labeling strategies for LFIA

The choice of nanoparticle label and its properties critically influence LFIA performance, with Au nanostructures being the classical option and UCNPs representing the emerging novel approach. AuNPs remain the most established labels in LFIA and are often used as a reference for assessing alternative materials. Conventionally, spherical AuNPs with a size of 30 to 40 nm are used, as smaller particles exhibit insufficient extinction cross sections [26]. The use of larger-sized Au nanostructures can increase the sensitivity of LFIA. Serebrennikova et al. [27] compared three different types of Au nanostructures as labels in LFIA for the detection of procalcitonin. The tested labels included various sizes of Au nanospheres (20 to 100 nm), Au nanostars (64 nm), and Au nanopopcorns (100 nm). The results indicated that hierarchical gold nanostructures, represented by Au nanopopcorns, provide the most intense color, improving the visual and quantitative detection. Compared to the LFIA with 20-nm Au nanospheres, the use of Au nanopopcorns resulted in a 5-fold lower LOD (0.1 ng/mL), which could be further decreased to 0.05 ng/mL using silver enhancement. The superior performance of large-sized Au nanostructures aligns with our findings, in which AuNSs (150 nm) yielded a significantly lower LOD compared to AuNPs (40 nm). More complex Au nanostructures, such as nanostars, nanopopcorns, and nanoflowers, might benefit from their three-dimensional configurations, which provide them greater chemical stability [28]; however, large Au nanostructures also tend to aggregate, as observed in our experiments detecting PSA in serum. In the literature, PSA was qualitatively detected in urine samples using AuNP-based LFIA with a cut-off value of 4 ng/mL [29]. A more advanced study by Di Nardo et al. [30] reported on a lateral flow biosensor for the detection of urinary PSA using gold nanoparticles functionalized with Staphylococcal protein A. The specific anti-PSA antibody was added separately (indirect competitive format), allowing fine-tuning of the assay (test line disappears when urine PSA exceeds the established cut-off). The LOD achieved with this system was 20 ng/mL. Commercial tests are also available, for example, from Elabscience, enabling the detection of PSA from whole blood and plasma with a cut-off value of 4 ng/mL [31].

UCNP-based LFIA represents an alternative to assays using conventional AuNP labels, with the performance strongly influenced by the UCNP surface modification. Bayoumy et al. [16] developed LFIA for the detection of cardiac troponin I using the commercial carboxyl-coated UCNPs, reaching the LOD of 30 pg/mL in the plasma samples. In a subsequent study, Raiko et al. [15] enhanced LFIA performance by implementing an automatic, low-cost actuator, which facilitated most of the LFIA procedure and thus reduced the manual labor. Moreover, commercial carboxyl-coated UCNPs used in the previous study were compared with in-house PAA-coated UCNPs with the same core. The results showed that PAA-coated UCNPs exhibited significantly lower background signals due to the reduced non-specific binding, resulting in an LOD of 1.5 pg/mL of cardiac troponin I. However, to the best of our knowledge, no comparison of PAA- and PEG-coated UCNPs as labels in LFIA has been conducted. Nevertheless, the superior performance of the PAA-coated UCNPs corresponds to observations presented in our study, where the PAA-coated UCNPs provided lower LODs compared to the PEG-coated ones. PAA-coated UCNPs were also successfully utilized for the sensitive LFIA detection of SARS-COV-2 nucleoprotein [17] and norfloxacin [32], confirming our findings that UCNP labels can enable the ultrasensitive LFIA detection of biomarkers.

Reports on PSA detection with UCNP-based LFIAs can also be found in the literature. A study by Hu et al. reported on the LFIA test for qualitative and quantitative PSA detection utilizing carboxyl-coated UCNPs labeled with anti-PSA antibodies *via* EDC/NHS chemistry [18]. The assay detected PSA in concentrations ranging from 0.1 to 100 ng/mL with an LOD of 0.1 ng/mL. In addition, Shen et al. introduced an enhanced centrifugation-assisted LFIA (ECLFIA) that integrates sample preparation, active flow control, washing, and signal amplification directly on a centrifugal disc [33]. This system involved only a nitrocellulose membrane and enabled the detection of PSA with an LOD of 28 pg/mL. Although the ECLFIA reaches high sensitivity, our UCNP-LFIA offers a more practical advantage by preserving the simplicity of a classic LFIA format without requiring complex centrifugation hardware. Yuan et al. reported an LFIA test for quantitative PSA detection using PAA-coated UCNPs conjugated with monoclonal anti-PSA antibody *via* EDC/NHS chemistry. The assay provided a linear range between 0 and 500 ng/mL and an LOD of 0.563 ng/mL, approximately one order of magnitude higher than our assay [34].

Also, other labeling strategies for PSA detection using LFIA were employed. For example, Kim et al. developed a semiquantitative LFIA based on SiO_2_@Au-Ag nanoparticle clusters, achieving a visual detection range of 0.3 to 10 ng/mL [35]. Cai et al. achieved quantitative detection of PSA using PAA-modified gold magnetic nanoparticles with an LOD of 0.17 ng/mL [36]. Bock et al. synthesized silica-coated CdSe@ZnS quantum dots *via* reverse microemulsion, which were able to achieve an LOD of 1 ng/mL for PSA [37]. Another approach involved fluorescent europium(III) chelate-doped nanoparticle reporters for quantitative detection of free PSA, reaching an LOD of 0.01 ng/mL [38]. The summary of analytical parameters of the discussed AuNP- and UCNP-based LFIAs is provided in Table S5.

The sensitivity of an LFIA assay is influenced not only by the choice of label but also by multiple other factors, such as the assay design, the detection system employed, the quality and affinity of the recognition elements, and even unique optimization strategies introduced during the development. Therefore, direct label comparison based only on the obtained LODs is challenging. In our study, PAA-modified UCNPs proved to be the most effective label for LFIA detection of PSA, successfully analyzing real serum samples with an LOD of 34 pg/mL (136 pg/mL considering the serum dilution). This LOD is comparable with the most sensitive assays, confirming the potential of PAA-modified UCNPs as labels for highly sensitive LFIAs for biomarker detection.

## Conclusions

This study focused on comparing various types of LFIA labels for protein biomarker detection in biological samples, including Au-based nanostructures—specifically AuNPs and AuNSs—and UCNPs with surface modification using PEG or PAA. First, all parameters of the LFIA were optimized using PEG-Er UCNP labels and HSA as the analyte, aiming to achieve the highest *S*/*B* ratio. Using optimal conditions, dip-stick and conjugate pad formats were compared. Among the tested Au-based and UCNP-based options, the best performance was achieved using the AuNSs and PAA-Tm UCNPs, both in the dip-stick format. To further improve the LFIA performance, we proposed using a piezo-driven dispenser for antibody deposition to establish test and control zones. The LODs of 76 and 32 pg/mL for the AuNS and PAA-Tm UCNP labels, respectively, clearly demonstrated the advantages of piezo-driven antibody dispensing for improving detection sensitivity. Subsequently, this approach was used to determine HSA in spiked urine samples, confirming the applicability of the assay for the analysis of complex biological samples. To evaluate the versatility of the assay, PSA determination in serum was carried out using the best-performing AuNS and PAA-Tm UCNP labels. LFIA with AuNSs achieved an LOD of 39 pg/mL; however, the particles tended to aggregate in the serum matrix, deteriorating the accuracy of the results. In contrast, LFIA with PAA-modified UCNPs showed an LOD of 34 pg/mL and excellent stability, with only minor aggregation in the serum. Finally, PSA concentrations were determined in spiked serum samples, and the data obtained using the PAA-Tm UCNPs showed a much stronger correlation with the reference levels than those obtained using the AuNSs. The results demonstrate that selecting a suitable label and surface modification is critical for reaching high assay sensitivity. The PAA-Tm UCNPs proved to be well-suited labels for sensitive LFIA detection of biomarkers, combining LODs in a clinically relevant range with high stability. Our results highlight UCNP labels as superior candidates for highly sensitive and robust LFIA assays, showcasing their potential for POC diagnostics.

## Supplementary Information

Below is the link to the electronic supplementary material.

Supplementary Material 1

## Data Availability

The data supporting the findings of this study are available within the paper and its Supplementary Information. Should any raw data files be needed, they are available from the corresponding author upon reasonable request.
