# Supplementary material for: Label matters: comparing gold nanoparticles and nanoshells with upconversion nanoparticles for quantitative lateral flow immunoassays
Source: Mikrochim Acta. 2026 Mar 31;193(4):287. doi: 10.1007/s00604-026-08015-5 (PMC13038764; doi:10.1007/s00604-026-08015-5)
Supplement: Supplementary file 1 — Supplementary Material 1 [file 604_2026_8015_MOESM1_ESM.pdf]

SUPPLEMENTARY INFORMATION

Label matters: Comparing gold nanoparticles and nanoshells with upconversion nanoparticles for quantitative lateral flow immunoassays

Eliška Macháčová[1], Jakub Máčala[1], Martin Kopecký[1], Saara Kuusinen[2], Tero Soukka[2], Zdeněk Farka[1,*]

[1]Department of Biochemistry, Faculty of Science, Masaryk University, Kamenice 5, 625 00 Brno, Czech Republic

[2]Department of Life Technologies/Biotechnology, Faculty of Technology, University of Turku, Kiinamyllynkatu 10, 20520 Turku, Finland

*Corresponding author. Email: farka@mail.muni.cz, Phone: +420 54949 7674

Table of contents

Materials and methods S-2

Preparation of labels S-2

UCNP-PEG-Ab labels S-2

UCNP-PAA-Ab labels S-3

AuNP-Ab labels S-4

AuNS-Ab labels S-4

Nanoparticle characterization S-5

Enzyme-linked immunosorbent assay S-6

Results and discussion S-7

Nanoparticle characterization S-7

Lateral flow immunoassay S-15

References S-21

Materials and methods

Preparation of labels

UCNP-PEG-Ab labels

Upconversion nanoparticles (UCNPs) composed of $NaYF_4:Yb^{3+},Er^{3+}$ or $NaYF_4:Yb^{3+},Tm^{3+}$ were synthesized using a high-temperature co-precipitation method according to our previously published protocol [1].

To prepare the alkyne-PEG-neridronate linker, 30 mg of neridronate (Merck, Germany) was dissolved in 128 µL of 1 M NaOH. Next, 398 µL of phosphate buffer (PB; 50 mM NaH_2PO_4/Na_2HPO_4; pH 7.4) was added, and the mixture was sonicated for 5 min. Then, 500 µL of 15 mM α-*N*-hydroxysuccinimide-ω-alkyne polyethylene glycol (3 kDa; Rapp Polymere, Germany) in PB was added, and the mixture was incubated for 2 h at room temperature (RT) with shaking, followed by overnight incubation at 4 °C with shaking. The mixture was then purified by dialysis against distilled water for 5 days (water exchange 3 times a day) using a Spectra/Por Float-A-Lyzer G2 dialysis tube (Carl Roth, Germany) with a molecular weight cut-off (MWCO) of 0.5–1 kDa. After the dialysis, the purified linker solution was transferred into a glass vial, lyophilized (FreeZone 2.5, Labconco, USA) for 24 h, and stored at 4 °C.

For the conjugation of UCNPs with the synthesized alkyne-PEG-neridronate linker, 10 mg of oleic acid-capped UCNPs in 500 µL of cyclohexane was mixed with 500 µL of 200 mM HCl and incubated for 40 min at 38 °C with shaking, followed by 30 min of sonication. This mediated the exchange of oleic acid surface ligands with Cl^- ions, allowing phase transfer to water. The upper organic phase was discarded, and excess acetone was added to the lower aqueous phase to precipitate the particles. The sample was then centrifuged at 1,000 × *g* for 1 h, the supernatant was discarded, and the UCNP pellet was dispersed in 500 µL of distilled water. Then, the dispersion was sonicated for 5 min. Subsequently, 3 mg of alkyne-PEG-neridronate linker was dissolved in 500 µL of distilled water and added to the UCNP dispersion. The mixture was incubated overnight at 38 °C with constant shaking. The prepared conjugates were purified by dialysis against distilled water containing 1 mM KF for 3 days (solution exchange 3 times a day) using Spectra/Por Float-A-Lyzer G2 dialysis tube (50 kDa MWCO).

The monoclonal anti-human serum albumin (HSA) antibody (AL-01, Exbio, Czech Republic) was modified using an NHS-dPEG$_8$-azide linker (Merck, Germany). First, the linker was dissolved in dimethylformamide (DMF) to obtain a 200 mM solution. Then, 1.96 µL of the solution was mixed with 200 µL of the antibody (2.5 mg/mL) in PB and 150 µL of PB. The mixture was incubated for 2 h at RT, and the reaction was stopped by adding 50 µL of 375 mM Tris (pH 7.5). The antibody-azide conjugate was purified using an Amicon Ultra centrifugal filter (10 kDa MWCO; Merck, Germany) and stored at 4 °C in PB at a final concentration of 1 mg/mL.

For the conjugation of UCNPs with azide-modified monoclonal anti-HSA antibody, 100 µL of Tris buffer (375 mM; pH 7.5) was mixed with an alkyne-PEG-neridronate-UCNP dispersion (10 mg in 1.4 mL of distilled water with 1 mM KF) and 20 µL of an aqueous solution of sodium ascorbate (20 mg/mL; Merck, Germany). The mixture was purged with argon for 40 min to remove any oxygen, and then 100 µL of antibody-azide in PB (1 mg/mL) was added, followed by another 10 min of argon purging. The click reaction was initiated by adding 10 µL of an aqueous $CuSO_4$ solution (6.25 mM). The dispersion was constantly purged, and after 10 min, the same amount of $CuSO_4$ solution was added again, followed by purging for an additional 2 h. The UCNP-antibody dispersion was then transferred to a Spectra/Por Float-A-Lyzer G2 dialysis tube (100 kDa MWCO) and dialyzed against dialysis buffer (50 mM Tris, 0.05% NaN_3, 1 mM KF; pH 7.5) at 4 °C for 3 days (buffer exchange 3 times a day). Purified UCNP-antibody conjugates were stored at 4 °C.

UCNP-PAA-Ab labels

Oleic acid-capped $NaYF_4$:Yb^{3+},Tm^{3+} (20% Yb^{3+}, 0.5% Tm^{3+}) UCNPs were synthesized using a previously published protocol [2]. The oleic acid was removed from the UCNP surface using nitrosonium tetrafluoroborate. First, 25 mg of oleic acid-capped UCNPs in toluene were diluted in cyclohexane to a concentration of 25 mg/mL. The mixture was combined with an equal volume of DMF and vortexed. The solution was transferred to a microtube containing 25 mg of nitrosonium tetrafluoroborate and mixed (1200 rpm) for 1 h at RT to replace the oleic acid with BF_4^- ions. The BF_4^--capped UCNPs were precipitated by adding 8 mL of chloroform, followed by centrifugation at 11,000 × *g* for 5 min. The supernatant was discarded, and the UCNP pellet was dispersed in 400 µL of DMF. The UCNPs were washed three more times by precipitating with 4 mL of chloroform, centrifuging at 11,000 × *g* for 5 min, and dispersing the pellet in 400 µL of DMF. After the last centrifugation, the UCNP pellet was dispersed in 240 µL of DMF. Large aggregates were removed by centrifuging at 2,500 × *g* for 3 min, and the BF_4^--capped UCNPs in the supernatant were transferred to another tube and diluted to a concentration of 50 mg/mL by adding DMF. To replace the BF_4^- ions with poly(acrylic acid) (PAA; 2 kDa; 50 wt. % in H_2O, Merck, Germany), the BF_4^--capped UCNPs were mixed with double the volume of 8.75% (w/v) PAA and 0.9 M 1,8-diazabicyclo[5.4.0]-7-undecene (DBU) in DMF, resulting in 16.7 mg/mL of UCNPs, 5.8% (w/v) PAA, 5.8% (w/v) H_2O, and 0.6 M DBU in the final reaction volume in DMF. The sample was incubated for 24 h at 60 °C with shaking at 1,400 rpm. The PAA-coated UCNPs were collected by centrifugation (20,238 × *g*, 30 min), and the supernatant was discarded. The UCNPs were washed twice with 1 mL of distilled water and once with 1 mL of 50 mM sodium borate buffer (pH 8), each time based on dispersing the pellet by bath sonication and centrifuging at 20,238 × *g* for 15 min. Finally, the UCNP pellet was dispersed in 250 µL of 50 mM sodium borate buffer (pH 8) and centrifuged at 2,500 × *g* for 3 min to remove large aggregates. The PAA-coated UCNPs in the supernatant were transferred into a new tube and stored at RT with slow rotation.

PAA-coated UCNPs were conjugated with anti-HSA or anti-prostate-specific antigen (PSA) (ab403, Abcam, UK) monoclonal antibodies. The antibodies were first transferred to a 10 mM PB (10 mM NaH_2PO_4/Na_2HPO_4; pH 7.4) using an Amicon Ultra centrifugal filter (10 kDa MWCO) to remove sodium azide. Subsequently, 1 mg of PAA-UCNPs was diluted with 20 mM MES buffer (pH 6.1) to a final concentration of 8 mg/mL. The dispersion was then

centrifuged for 20 min at 20,238 × *g*. The supernatant was discarded, and the UCNP pellet was redispersed in 125 µL of MES buffer by sonication and vortexing. Afterward, 2.5 µL of sulfo-NHS (1.56 M in MES) was added to the UCNP dispersion, followed by 2.5 µL of EDC (0.78 M in MES). The mixture was incubated for 45 min with rotation. After incubation, the mixture was centrifuged for 7 min at 20,238 × *g*. The supernatant was then removed, and the pellet was redispersed in 168 µL of MES buffer, followed by another 7 min of centrifugation at 20,238 × *g*. The supernatant was discarded, and the pellet was redispersed in 25 µL of 100 mM MES buffer (pH 6.1), followed by the addition of 50 µg of antibody to yield a total volume of 125 µL. The mixture was vortexed and incubated for 2.5 h at RT with shaking (1400 rpm). After incubation, 3.21 µL of 2 M glycine (pH 11) was added to the mixture to quench the reaction, followed by 30 min of incubation with rotation. Subsequently, the mixture was centrifuged three times at 20,238 × *g* for 10 min. The supernatant was discarded after each centrifugation, and the pellet was redispersed in 500 µL of 10 mM Tris with 0.1% Tween 20 (pH 8). After the last centrifugation, the pellet was redispersed in 125 µL of storage buffer (5 mM Tris, 0.05% Tween 20, 0.5% BSA, 0.05% NaN_3, 5% trehalose, 5% ethylene glycol; pH 8.5) and stored at 4 °C.

AuNP-Ab labels

The BioReady 40-nm carboxyl-coated gold nanoparticles (AuNPs; nanoComposix, USA) were conjugated with an anti-HSA monoclonal antibody. First, the dispersion of AuNPs (optical density of 20) was sonicated and vortexed, and 0.5 mL was added to a microcentrifuge tube. Subsequently, 10 µL of EDC (10 mg/mL in water) and 20 µL of sulfo-NHS (10 mg/mL in water) were added to the nanoparticle dispersion, and the dispersion was incubated for 30 min at RT with rotation. After the incubation, the dispersion was centrifuged twice at 3,800 × *g* for 10 min. The supernatant was discarded after each centrifugation, and the pellet was redispersed in 0.5 mL of reaction buffer (5 mM K_2HPO_4/KH_2PO_4, 0.5% PEG (6 kDa); pH 7.4) by sonication and vortexing. Subsequently, 25 µg of the antibody was added, and the mixture was incubated for 1 h with rotation. Then, 15 µL of 5 M Tris in water was added to deactivate the remaining reactive NHS esters. The mixture was incubated for an additional 10 min with rotation and then purified three times by centrifugation at 3,800 × *g* for 10 min. After the first and the second centrifugation, the supernatant was removed, and the pellet was redispersed in 500 µL of reaction buffer. After the third centrifugation, the pellet was redispersed in 500 µL of conjugate diluent (25 mM NaH_2PO_4/Na_2HPO_4, 75 mM NaCl, 0.5% BSA, 0.5% casein, 1% Tween 20, 0.05% NaN_3; pH 8). The prepared conjugate dispersion was stored at 4 °C.

AuNS-Ab labels

The BioReady 150-nm carboxyl-coated gold nanoshells (AuNSs; nanoComposix, USA) were conjugated with anti-HSA or anti-PSA monoclonal antibodies. The protocols for both conjugations were identical. The dispersion of AuNSs (optical density of 20) was sonicated and vortexed, and 0.5 mL of the dispersion was pipetted into a microcentrifuge tube. Then, 4 µL of EDC (10 mg/mL in water) and 8 µL of sulfo-NHS (10 mg/mL in water) were added to the dispersion. The mixture was then incubated at RT for 30 min with rotation and centrifuged twice at 2,000 × *g* for 5 min. The supernatant was discarded, and the pellet was redispersed by sonication and vortexing in 0.5 mL of reaction buffer. After the second redispersion, 15 µg of

the antibody was added, and the mixture was incubated with rotation for 1 h at RT. Subsequently, 30 μL of 5 M Tris in water was added to deactivate the remaining reactive NHS esters, followed by 10 min of incubation. Next, the dispersion was centrifuged three times at 2,000 × *g* for 5 min. The first two centrifugations were followed by redispersion in 0.5 mL of reaction buffer. After the last centrifugation, the pellet was thoroughly dispersed by sonication and vortexing in 0.5 mL of conjugate diluent and subsequently stored at 4 °C.

Nanoparticle characterization

The nanoparticles were characterized by transmission electron microscopy (TEM), dynamic light scattering (DLS), and emission spectra measurement. For TEM analysis, oleic acid-capped UCNPs dispersed in cyclohexane or Au-based nanostructures dispersed in water were dispensed onto a copper grid covered by a continuous carbon layer. The grid was dried for 5 min at RT, and the nanoparticles were imaged using a TALOS microscope equipped with a Falcon camera (ThermoFisher Scientific, Czech Republic) at an acceleration voltage of 200 kV. The size of individual UCNPs was analyzed using ImageJ software (National Institutes of Health, USA).

DLS measurements of the hydrodynamic diameters of particles and their bioconjugates were performed using a Zetasizer Nano ZS (Malvern, UK). Particles were diluted 500× in either cyclohexane (oleic acid-capped UCNPs) or TBS (other particles and bioconjugates). The measurements were performed at 25 °C using a scattering angle of 173°.

To measure the emission spectra, oleic acid-capped UCNPs were diluted 250× in cyclohexane and transferred into a rectangular quartz cuvette, which was placed in an in-house-made measurement cell. A 980-nm laser PSU-III-LED (Changchun New Industries Optoelectronics Technology, China) was used for excitation, and the emission was recorded with an AvaSpec ULS2048XL-RS-EVO-UA spectrometer using the AvaSoft 8.1 software (Avantes, Netherlands). The integration time of 2 s was used for all measurements.

For the absorption spectra measurements, gold nanostructures were diluted to an OD of ~0.8 in water, and the dispersion was analyzed using a Specord 210 Plus spectrophotometer (Analytik Jena, Germany).

An upconversion-linked immunosorbent assay was performed to confirm that UCNPs were successfully conjugated with antibodies. A high-binding microtiter plate (Greiner Bio-One, Austria) was coated with varying concentrations of goat anti-mouse polyclonal antibody (115-005-003; Jackson ImmunoResearch, UK) diluted in carbonate buffer at 100 μL/well. The plate was incubated for 16 h at 4 °C and then washed four times with a washing buffer. The remaining binding sites on the plate were blocked with 20% (v/v) SuperBlock TBS (Thermo Fisher Scientific, USA) in washing buffer at 200 μL/well; the microtiter plate was incubated for 1 h with shaking at 300 rpm using a Titramax 101 shaker (Heidolph, Germany) and then washed four times with washing buffer. Afterward, mouse monoclonal antibody-conjugated labels diluted to various concentrations in assay buffer were added at 100 μL/well; the plate was incubated for 1 h with shaking at 300 rpm, followed by four washes with washing buffer and drying at RT.

The plate was analyzed using the Upcon S-Pro reader (Labrox, Finland) with a 980-nm laser and a 976/60 nm excitation filter. Detection of Er-doped labels involved a D800 dichroic mirror and a 540/60 nm emission filter, while Tm-doped labels were detected with a D900 dichroic mirror and an 810/40 nm emission filter. The integration time, emission spot size, and relative laser power were set to 500 ms, 4 mm, and 100%, respectively. The wells were raster-scanned with 64 points (8 × 8 grid) at a spacing of 2.1 mm between the first and the last row. A truncated average was calculated for each well by excluding 25% of the lowest and highest values. The average signals and the standard deviations were calculated from 3 replicate wells.

Enzyme-linked immunosorbent assay

To conduct an enzyme-linked immunosorbent assay (ELISA), a high-binding microtiter plate was coated with 1 µg/mL of a mouse anti-PSA monoclonal antibody in carbonate buffer at 100 µL/well. The plate was incubated for 16 h at 4 °C and then washed four times with washing buffer. The remaining binding sites on the plate were blocked with 20% (v/v) SuperBlock TBS in washing buffer at 200 µL/well; the microtiter plate was incubated for 1 h with shaking at 300 rpm using a Titramax 101 shaker and then washed four times. Afterward, calibrators (PSA diluted in 25% plasma in assay buffer) or spiked plasma samples diluted correspondingly in assay buffer were added at 100 µL/well. The plate was incubated for 1 h with shaking at 300 rpm, followed by four washes. Subsequently, biotinylated goat anti-PSA polyclonal antibody (BAF1344, R&D Systems, USA; 0.25 µg/mL in assay buffer) was added at 100 µL/well. The plate was incubated for 1 h with shaking at 300 rpm, followed by four washes. Finally, streptavidin-horseradish peroxidase conjugate (ab7403, Abcam, UK; 5 ng/mL in assay buffer) was added to the plate at 100 µL/well. After 1 h of incubation with shaking (300 rpm) and four washing cycles, TMB-Complete 2 solution (TestLine, Czech Republic) was added to the plate at 100 µL/well. The increase in absorbance at 652 nm was monitored over time using a Synergy 2 reader (BioTek Instruments, USA). When the highest value reached 0.8, sulfuric acid (1 M) was added at 100 µL/well, and the plate was measured again at 450 nm. All buffers used in ELISA were free from NaN_3.

Results and discussion

Nanoparticle characterization

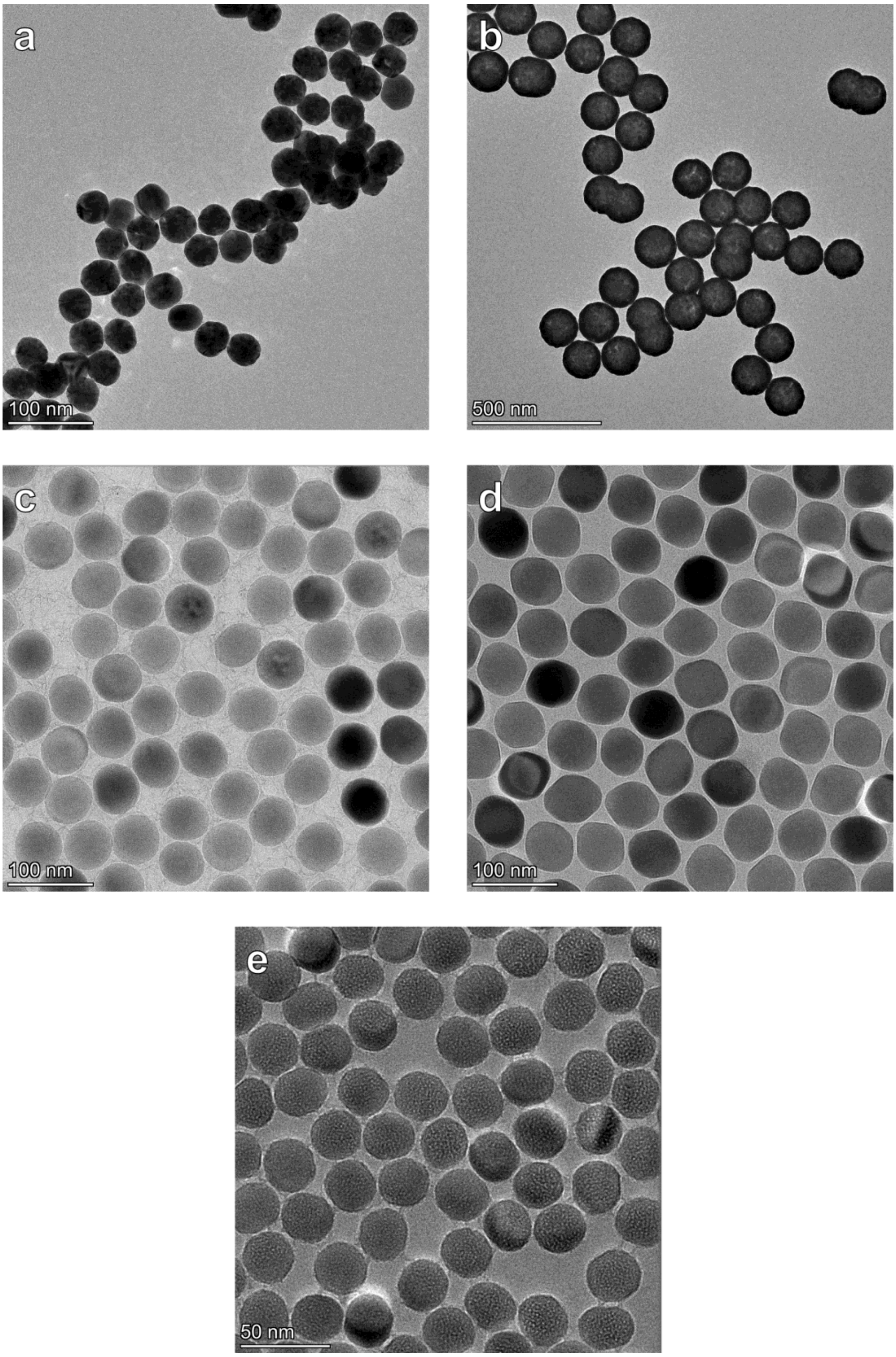

Fig. S1 TEM images of stock Au nanostructures and oleic acid-capped UCNPs. **a** AuNPs, **b** AuNSs, **c** $NaYF_4:Yb^{3+},Er^{3+}$ (PEG-Er UCNPs), **d** $NaYF_4:Yb^{3+},Tm^{3+}$ (PEG-Tm UCNPs), and **e** $NaYF_4:Yb^{3+},Tm^{3+}$ (PAA-Tm UCNPs).

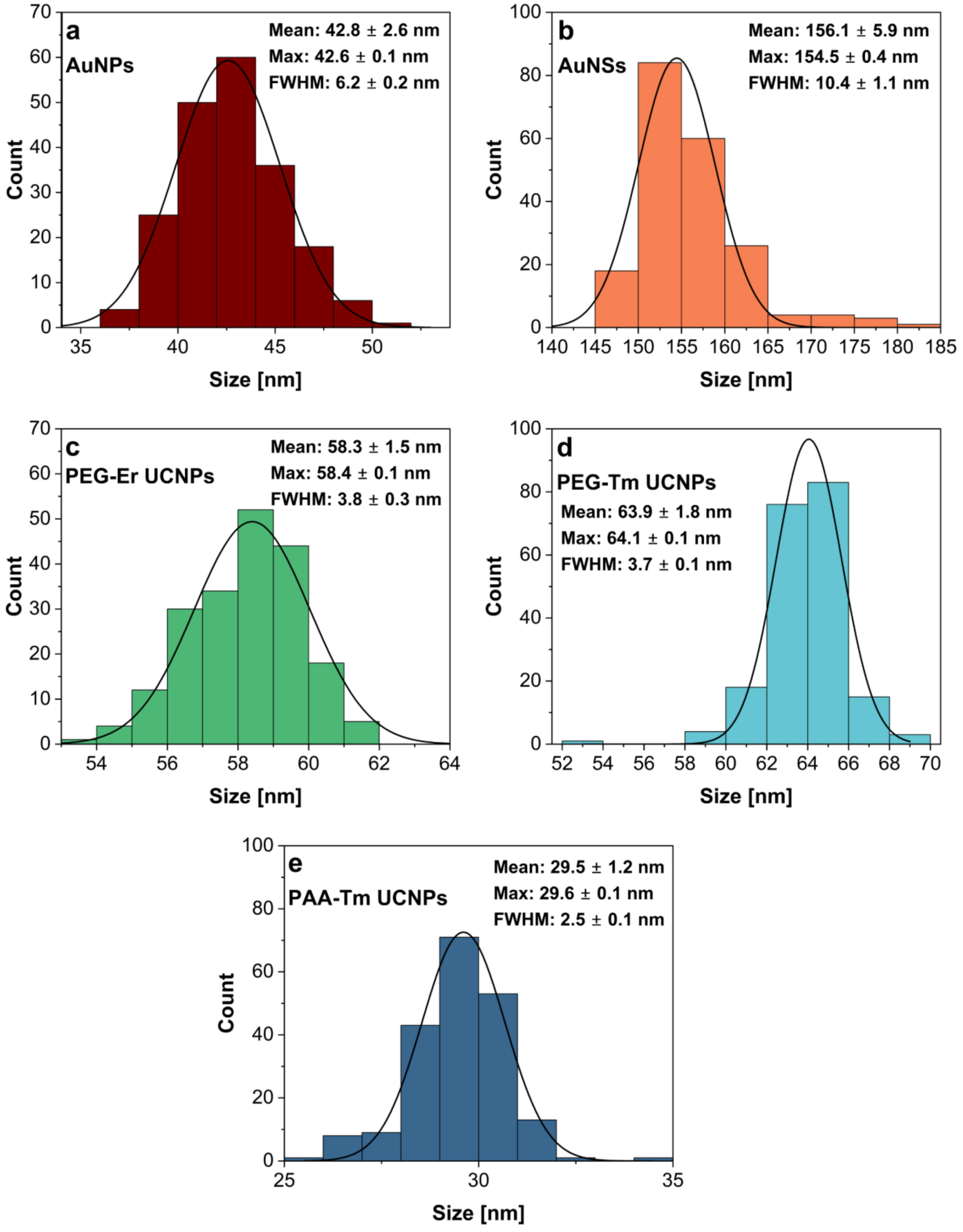

Fig. S2 Histograms of particle sizes (n = 200) with Gaussian fits evaluated from TEM images of stock Au nanostructures and oleic acid-capped UCNPs. **a** AuNPs, **b** AuNSs, **c** $NaYF_4:Yb^{3+},Er^{3+}$ (PEG-Er UCNPs), **d** $NaYF_4:Yb^{3+},Tm^{3+}$ (PEG-Tm UCNPs), and **e** $NaYF_4:Yb^{3+},Tm^{3+}$ (PAA-Tm UCNPs).

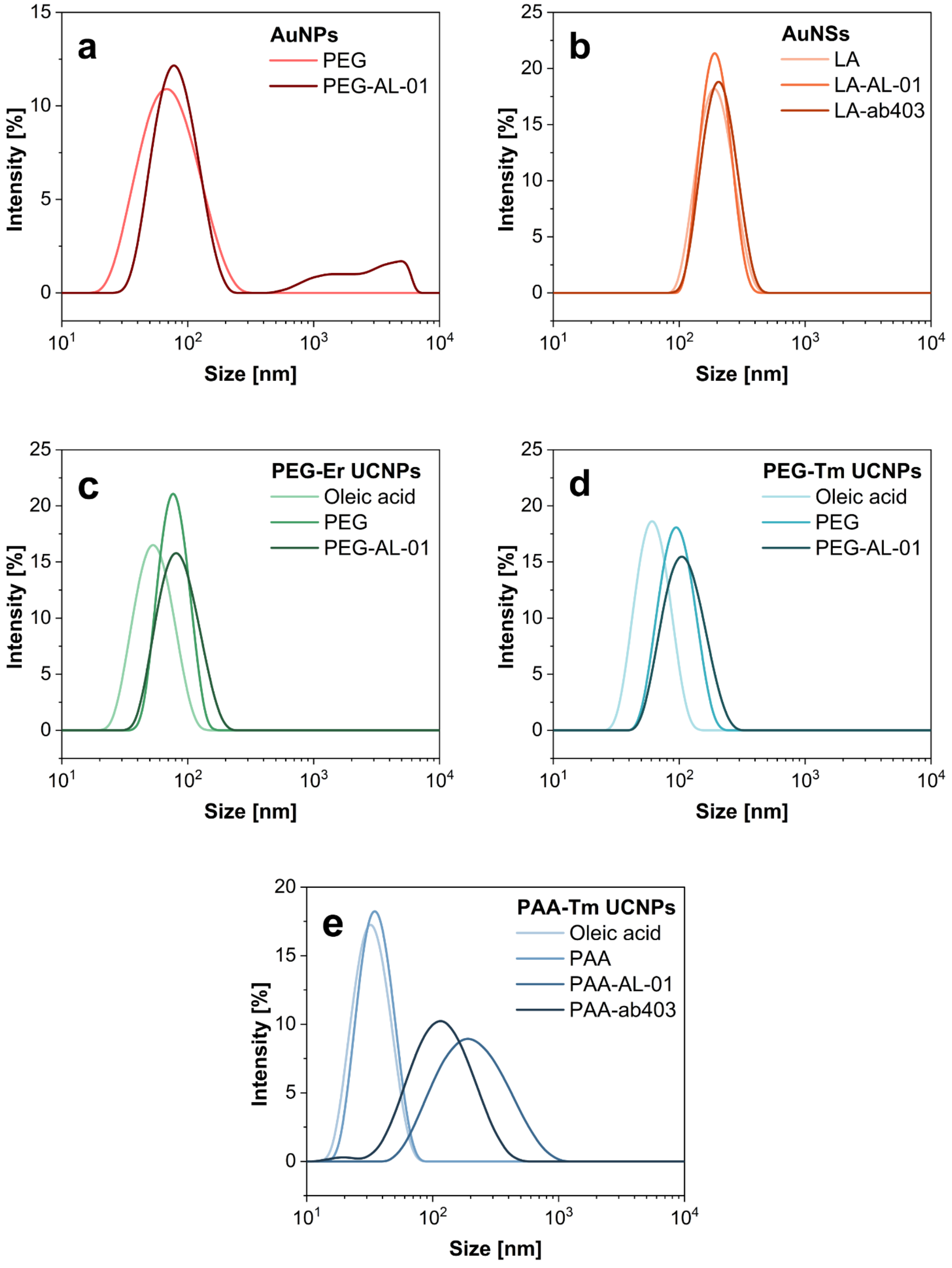

Fig. S3 DLS particle size distributions of **a** AuNPs, **b** AuNSs, **c** PEG-Er UCNPs, **d** PEG-Tm UCNPs, and **e** PAA-Tm UCNPs, their modifications, and conjugates with different monoclonal antibodies (AL-01 or ab403). The measured data points were connected using a B-spline function. (LA – lipoic acid)

Table S1 Particle size (Z-average) and polydispersity index (PdI) values obtained from the DLS measurements of the Au- and UCNP-based nanomaterials and their modifications.

Particle	Surface modification	Z-average [nm]	PdI
**AuNP**	**PEG**	61 ± 1	0.203
	**PEG-AL-01**	92 ± 1	0.386
**AuNS**	**LA**	194 ± 2	0.184
	**LA-AL-01**	190 ± 2	0.048
	**LA-ab403**	201 ± 1	0.057
**PEG-Er UCNP**	**OA**	51.0 ± 0.1	0.077
	**PEG**	74.3 ± 0.6	0.037
	**PEG-AL-01**	77.9 ± 0.8	0.150
**PEG-Tm UCNP**	**OA**	59.1 ± 0.5	0.036
	**PEG**	91.9 ± 0.7	0.069
	**PEG-AL-01**	101.3 ± 0.3	0.110
**PAA-Tm UCNP**	**OA**	30.7 ± 0.1	0.095
	**PAA**	33.7 ± 0.1	0.079
	**PAA-AL-01**	168 ± 2	0.256
	**PAA-ab403**	100 ± 2	0.242

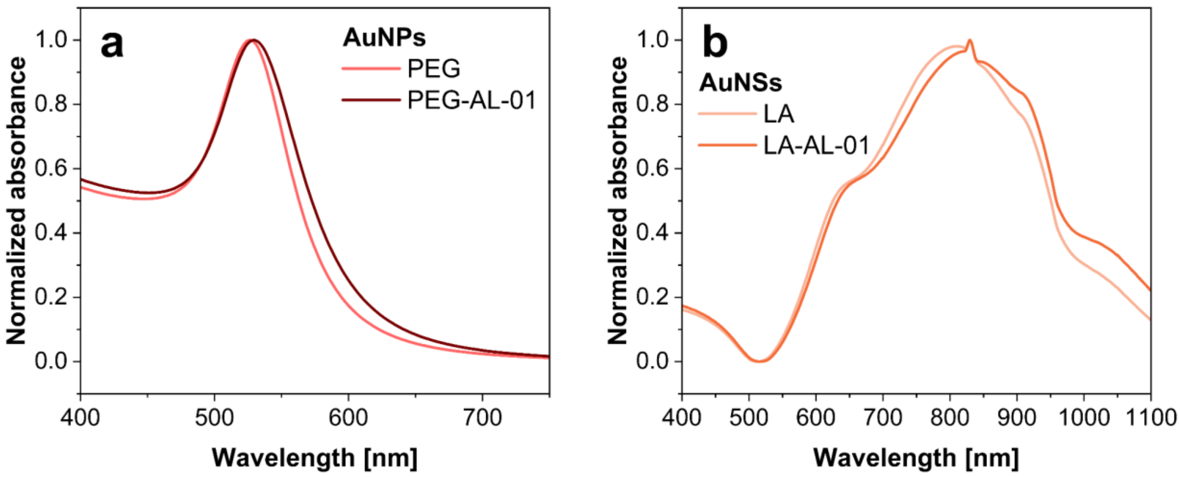

Fig. S4 Absorption spectra of bare **a** AuNPs and **b** AuNSs and their conjugates with antibodies. The red shifts indicate the addition of protein mass to the particle surface, confirming the successful formation of UCNP-antibody conjugates.

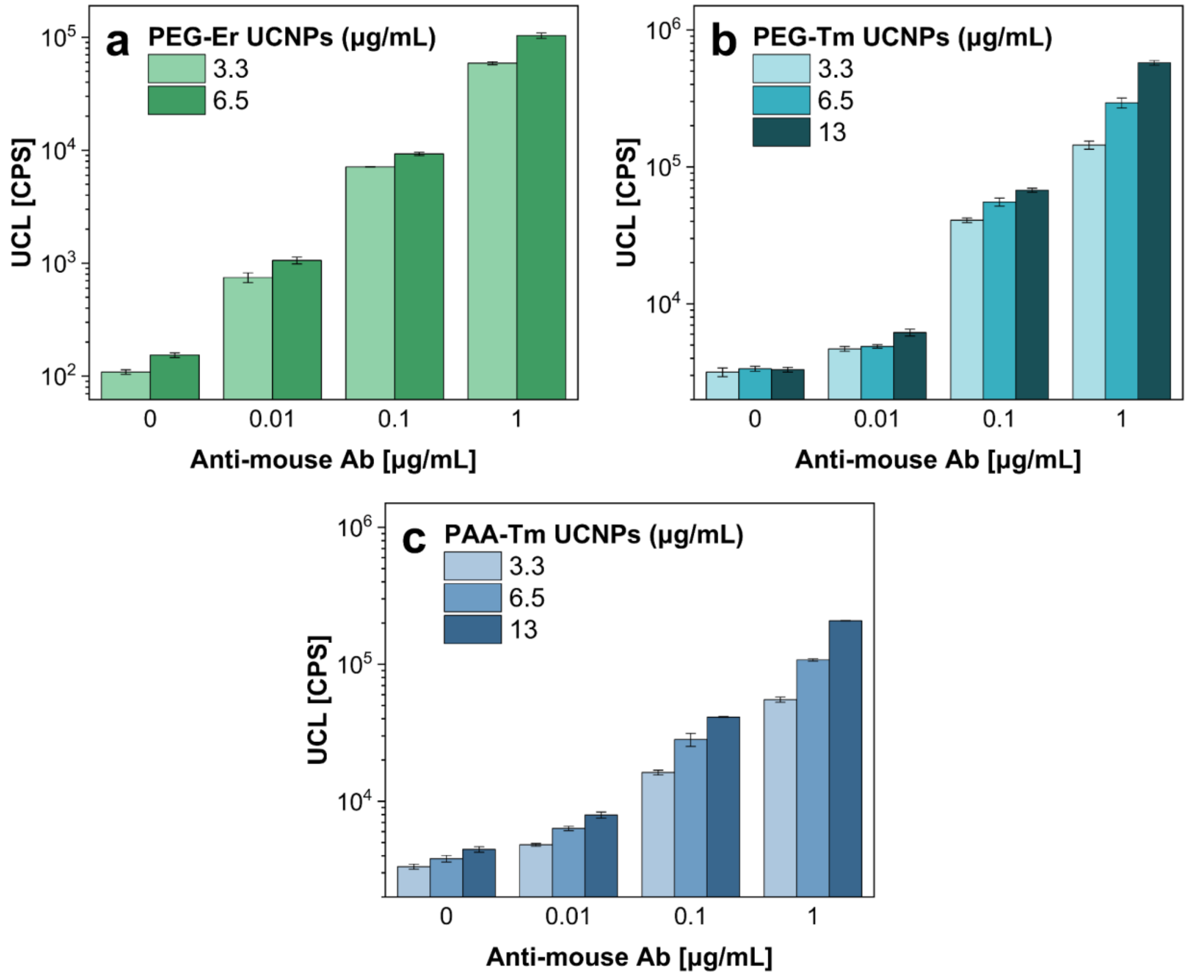

Fig. S5 Upconversion-linked immunosorbent assay for the verification of successful conjugation of UCNPs with mouse monoclonal antibodies. **a** PEG-Er UCNPs (AL-01), **b** PEG-Tm UCNPs (AL-01), and **c** PAA-Tm UCNPs (ab403). Error bars represent standard deviations. A specific signal increase with increasing anti-mouse antibody concentration was observed with all tested conjugates, confirming the presence of anti-analyte antibodies on the particle surface. The negative control, lacking anti-mouse antibody in the microtiter plate wells, was used to determine the background signal originating from non-specific adsorption of UCNP conjugates.

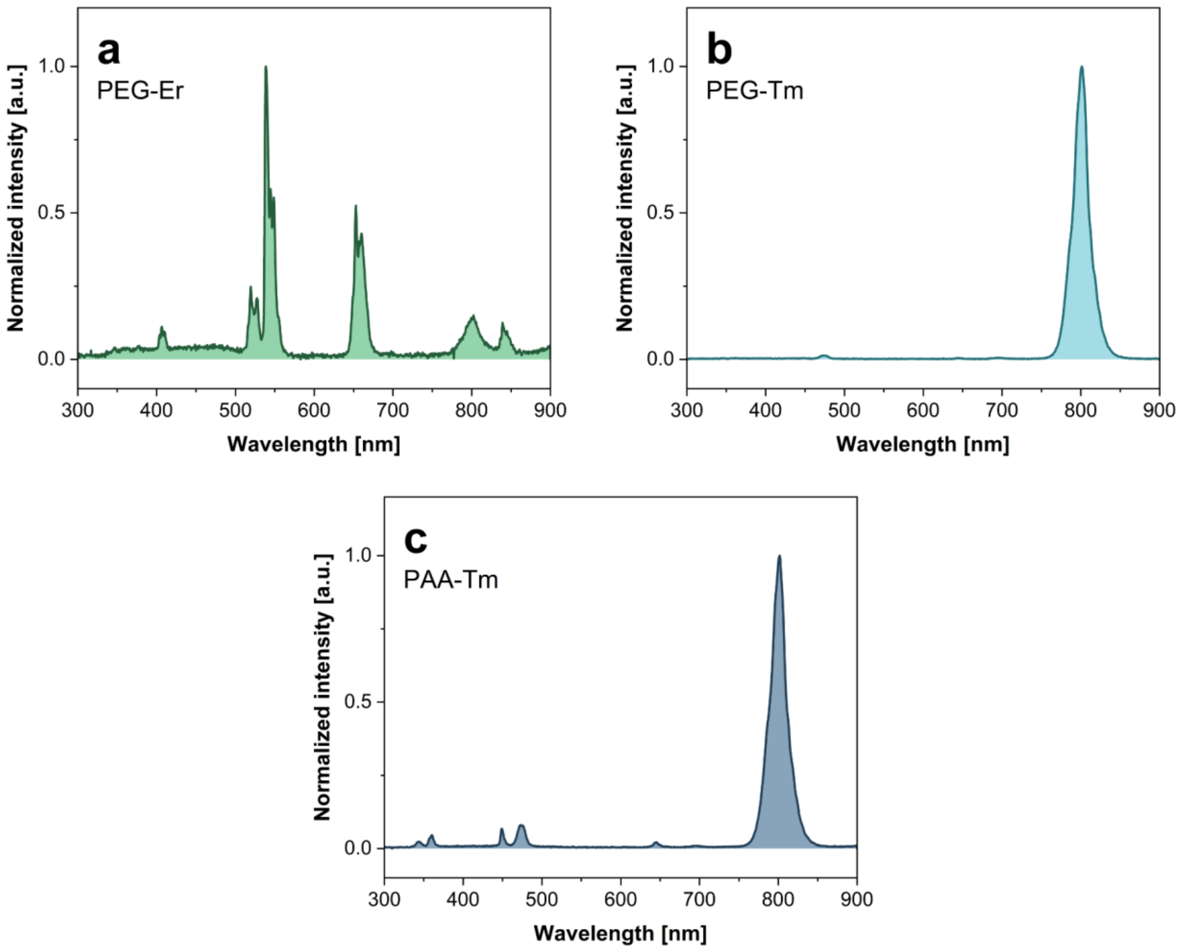

Fig. S6 Emission spectra of oleic acid-capped UCNPs under 980-nm excitation. **a** $NaYF_4:Yb^{3+},Er^{3+}$ (PEG-Er UCNPs), **b** $NaYF_4:Yb^{3+},Tm^{3+}$ (PEG-Tm UCNPs), and **c** $NaYF_4:Yb^{3+},Tm^{3+}$ (PAA-Tm UCNPs).

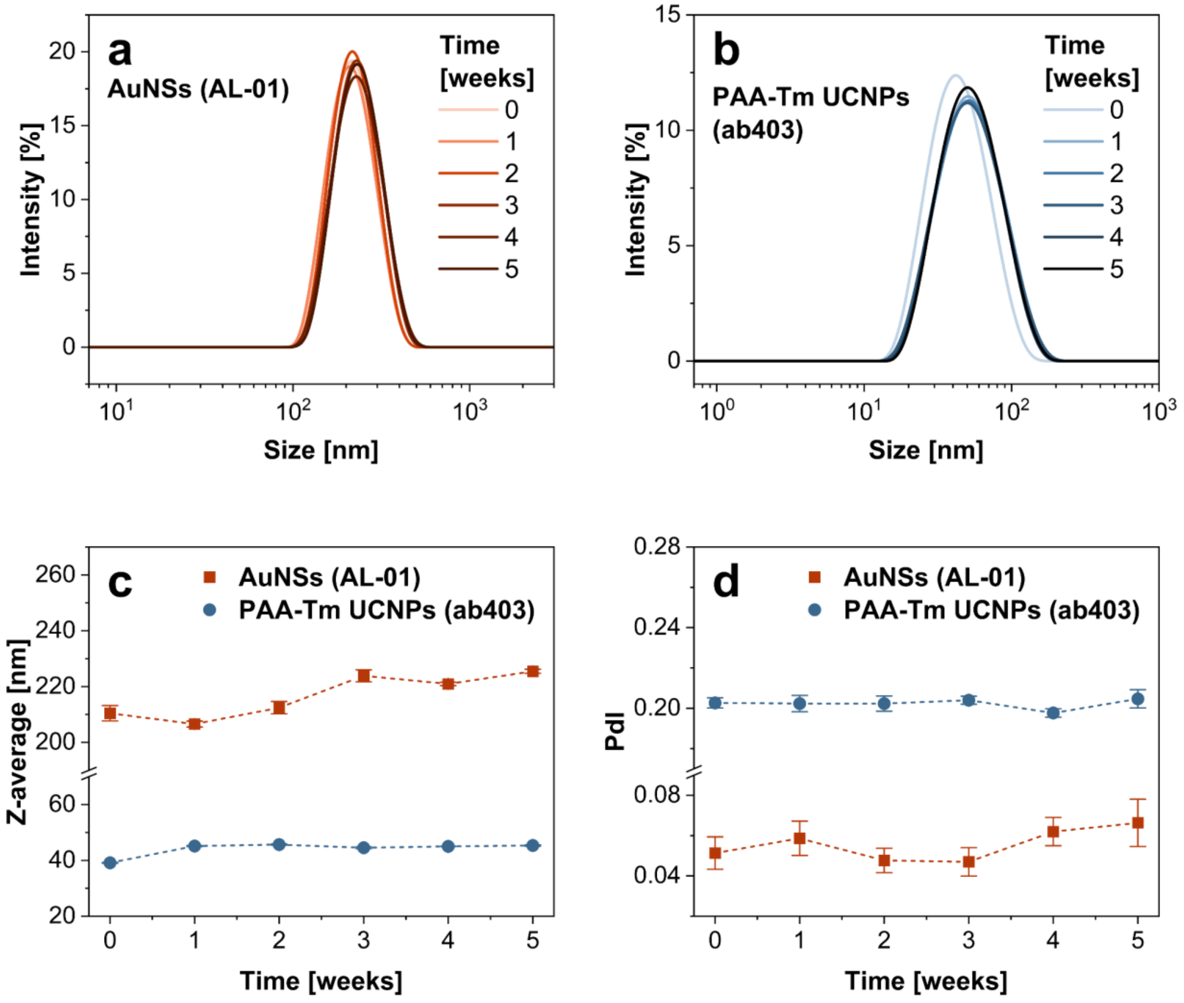

Fig. S7 DLS particle size distributions of **a** AuNSs (AL-01) and **b** PAA-Tm UCNPs (ab403) during 5 weeks of storage after surface modification, and the respective **c** Z-average and **d** PdI values. No significant aggregation was observed during the experiment, confirming that the particles remained colloidally stable.

Lateral flow immunoassay

Table S2 Analytical parameters of different LFIA approaches for HSA detection.

Label type	Assay type	LOD [ng/mL]	EC_{20}–EC_{80} [ng/mL]	R^2
Au NPs	Conjugate pad	7.2	18–140	0.99
Au NSs	Conjugate pad	1.8	12–210	0.99
Au NPs	Dip-stick	3.3	9.0–120	0.99
Au NSs	Dip-stick	0.94	1.7–18	0.99
Er-PEG UCNPs	Conjugate pad	160	800–13000	0.99
Tm-PEG UCNPs	Conjugate pad	1.3	160–2300	0.99
Tm-PAA UCNPs	Conjugate pad	0.2	550–330000	0.99
Er-PEG UCNPs	Dip-stick	2.2	120–2800	0.99
Tm-PEG UCNPs	Dip-stick	0.6	250–4300	0.99
Tm-PAA UCNPs	Dip-stick	0.12	3.9–42	0.99

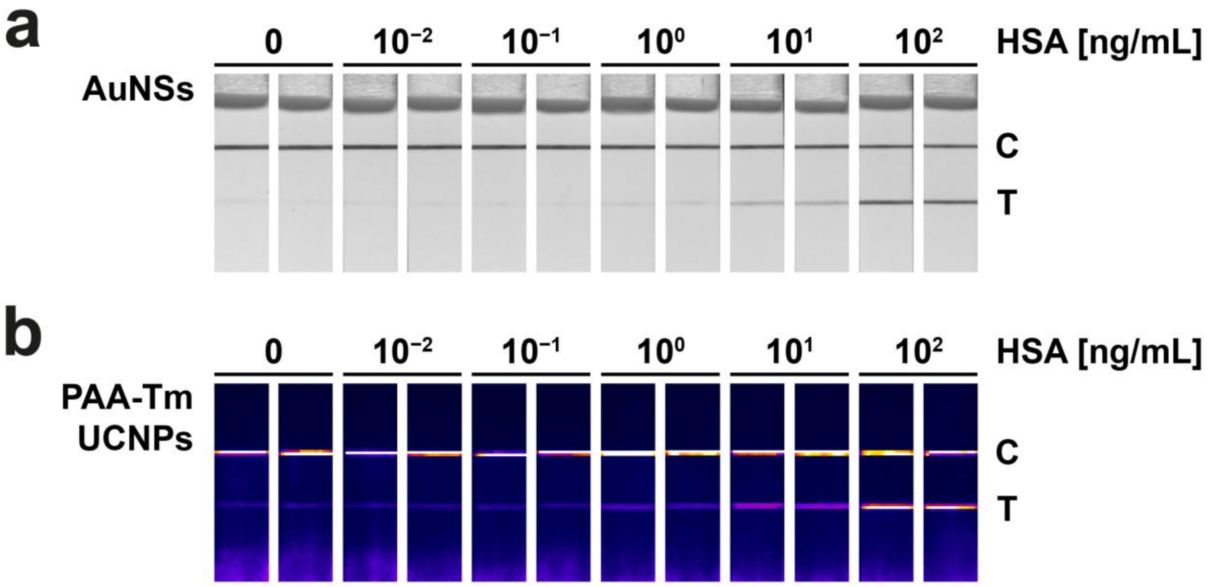

Fig. S8 Images of LFIA strips after the detection of HSA. Dip-stick approach with piezo-driven line deposition of antibodies with labels based on **a** AuNSs (optical density of 20) imaged using a densitometer and **b** PAA-Tm UCNPs (concentration of 6.5 µg/mL) visualized using an upconversion scanner.

Table S3 Analytical parameters of LFIA assays for HSA and PSA detection with different label types.

Label type	Analyte	Matrix	LOD [pg/mL]	EC_{20}–EC_{80} [ng/mL]	R^2
Au NPs	HSA	Buffer	1100	5.3–81	0.99
Au NSs	HSA	Buffer	76	6.2–76	0.99
Au NSs	HSA	Urine A	580	9.0–140	0.99
Au NSs	HSA	Urine B	150	9.7–180	0.99
Er-PEG UCNPs	HSA	Buffer	5700	65–630	0.99
Tm-PEG UCNPs	HSA	Buffer	270	60–350	1.00
Tm-PAA UCNPs	HSA	Buffer	32	4.5–72	0.99
Tm-PAA UCNPs	HSA	Urine A	63	5.5–88	0.99
Tm-PAA UCNPs	HSA	Urine B	200	7.2–120	0.99
Au NSs	PSA	Serum	39	0.88–14	0.99
Tm-PAA UCNPs	PSA	Serum	34	0.46–2.7	0.95
Tm-PAA UCNPs	PSA	Female plasma	80	1.5–13	0.99
Tm-PAA UCNPs	PSA	Male plasma	64	2.9–33	0.99

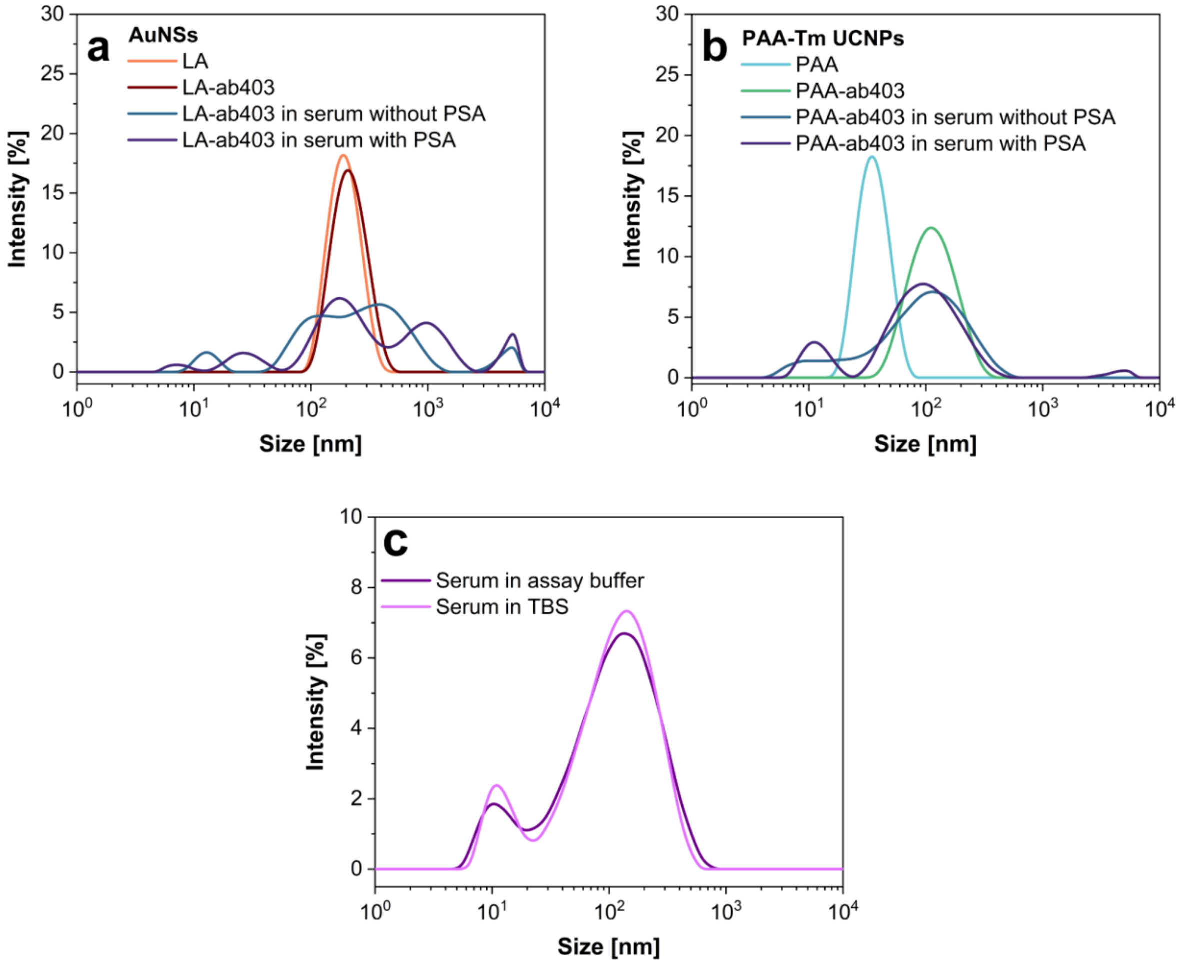

Fig. S9 DLS particle size distributions of conjugates of anti-PSA antibody (ab403) with **a** AuNSs and **b** PAA-Tm UCNPs in serum with and without PSA. **c** Particle size distribution of serum in different buffers.

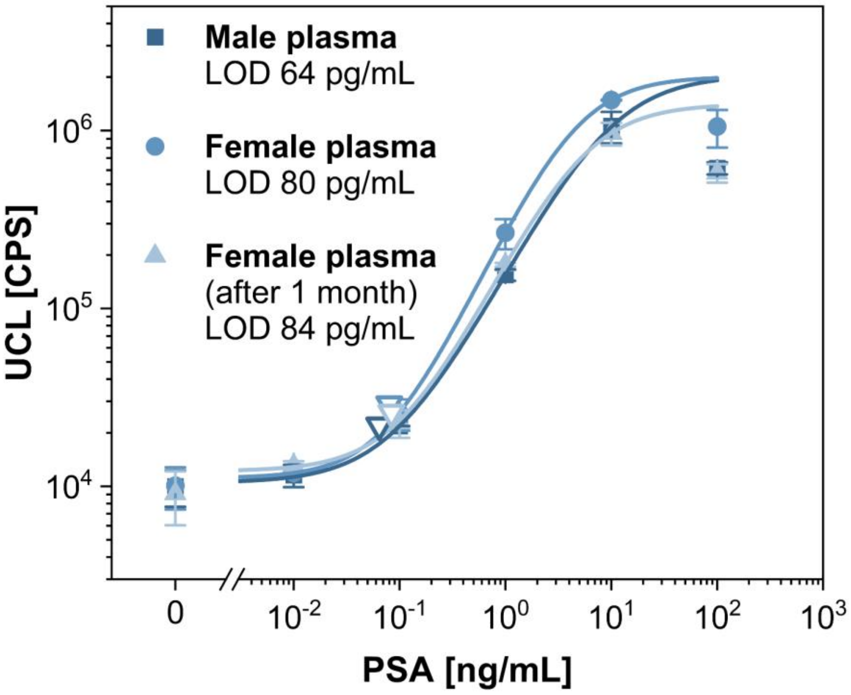

Fig. S10 Calibration curves for PSA detection in plasma using LFIA with PAA-Tm UCNP labels. Male and female plasma were used to investigate the matrix effect on the assay performance. Moreover, the female plasma was used for calibration after 1 month to examine the long-term storage stability of LFIA strips and PAA-Tm UCNP labels. Error bars represent standard deviations, and empty triangles indicate the LODs.

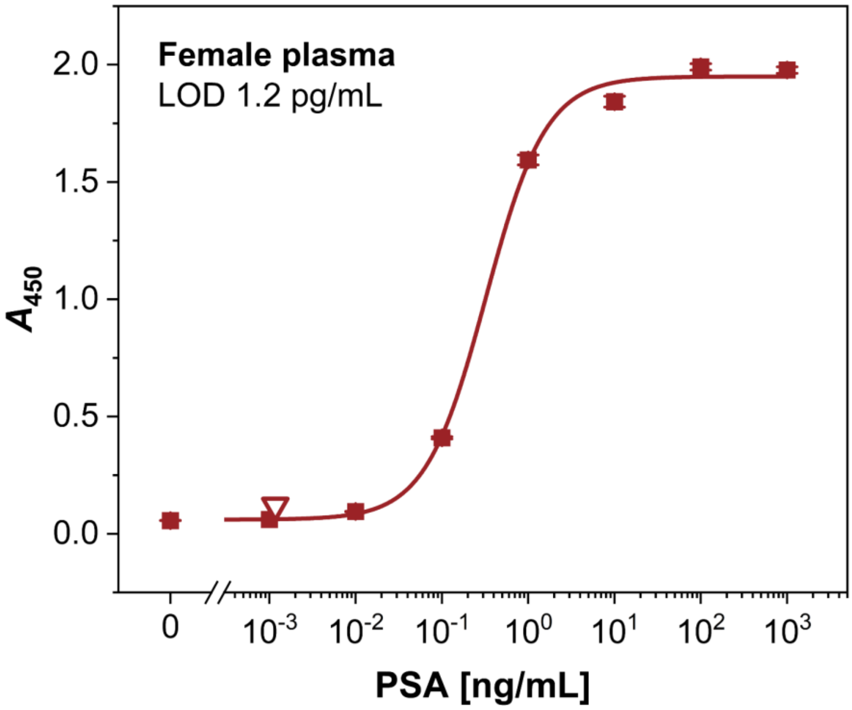

Fig. S11 Calibration curve for PSA detection in female plasma using ELISA. Error bars represent standard deviations, and an empty triangle indicates the LOD.

Table S4 Determination of PSA in spiked female plasma samples by LFIA with PAA-Tm UCNPs and by ELISA.

Spiked [ng/mL]	Dilution factor	LFIA with PAA-Tm UCNPs			ELISA		
		Found in diluted sample [ng/mL]	Found in original sample [ng/mL]	Recovery [%]	Found in diluted sample [ng/mL]	Found in original sample [ng/mL]	Recovery [%]
10	10	1.00 ± 0.07	10.0 ± 0.7	100 ± 7	1.2 ± 0.1	12 ± 1	116 ± 10
50	40	1.22 ± 0.02	48.9 ± 0.7	98 ± 1	1.46 ± 0.07	58 ± 3	116 ± 5
100	40	2.6 ± 0.3	103 ± 11	103 ± 11	2.7 ± 0.4	106 ± 14	106 ± 14
200	40	5.2 ± 0.6	208 ± 22	104 ± 11	4.9 ± 0.1	194 ± 6	97 ± 3

Table S5 Comparison of analytical parameters of different AuNP- and UCNP-based LFIAs.

**Label**	**Analyte**	**LOD [pg/mL]**	**Sample type**	**Working range**	**Reference**
Au nanostars and nanopopcorns	Procalcitonin	100	Buffer	0.5–10 ng/mL	[3]
AuNPs	PSA	Qualitative	Serum/plasma	N/A	[4]
AuNPs	PSA	20000	Urine	37–420 ng/mL	[5]
AuNPs	PSA	4000	Whole blood	N/A	[6]
COOH-UCNPs	cTnI	30	Plasma	37–9365 pg/mL	[7]
PAA-UCNPs	cTnI	1.5	Plasma	N/A	[8]
PAA-UCNPs	SARS-CoV-2 nucleoprotein	10	Buffer	10–10^6 pg/mL	[9]
PAA-UCNPs	Norfloxacin	2500	Milk	N/A	[10]
COOH-UCNPs	PSA	100	Serum/fingertip blood	0.1–100 ng/mL	[11]
PAA-UCNPs	PSA	563	Fetal bovine serum	0.1–500 ng/mL	[12]
AuNP	PSA	28	Blood	N/A	[13]
SiO_2@Au-Ag NPs	PSA	200	Serum	0.3–300 ng/mL	[14]
Magnetic AuNPs	PSA	170	Serum	N/A	[15]
QDs	PSA	1000	Buffer	N/A	[16]
Eu III NPs	PSA	10	Serum	0.1–25 ng/mL	[17]
Tm-PAA UCNPs	PSA	34	Serum	0.46–2.74 ng/mL	This work

References

1. Hlaváček A, Farka Z, Mickert MJ, et al (2022) Bioconjugates of photon-upconversion nanoparticles for cancer biomarker detection and imaging. Nat Protoc 17:1028–1072. https://doi.org/10.1038/s41596-021-00670-7

2. Palo E, Tuomisto M, Hyppänen I, et al (2017) Highly uniform up-converting nanoparticles: Why you should control your synthesis even more. J Lumin 185:125–131. https://doi.org/10.1016/j.jlumin.2016.12.051

3. Serebrennikova K, Samsonova J, Osipov A (2018) Hierarchical Nanogold Labels to Improve the Sensitivity of Lateral Flow Immunoassay. Nanomicro Lett 10:24. https://doi.org/10.1007/s40820-017-0180-2

4. Ashida S, Yamasaki I, Kawada C, et al (2021) Evaluation of a rapid one-step PSA test for primary prostate cancer screening. BMC Urol 21:135. https://doi.org/10.1186/s12894-021-00903-7

5. Di Nardo F, Occhipinti S, Gontero P, et al (2020) Detection of urinary prostate specific antigen by a lateral flow biosensor predicting repeat prostate biopsy outcome. Sens Actuators B Chem 325:128812. https://doi.org/10.1016/j.snb.2020.128812

6. Elabscience (Accessed 2025-11-12) Human Prostate Specific Antigen Lateral Flow Assay Kit. https://www.vetassay-elab.com/p-human_prostate_specific_antigen_lateral_flow_assay_kit-2151.html

7. Bayoumy S, Martiskainen I, Heikkilä T, et al (2021) Sensitive and quantitative detection of cardiac troponin I with upconverting nanoparticle lateral flow test with minimized interference. Sci Rep 11:18698. https://doi.org/10.1038/s41598-021-98199-y

8. Raiko K, Nääjärvi O, Ekman M, et al (2024) Improved sensitivity and automation of a multi-step upconversion lateral flow immunoassay using a 3D-printed actuation mechanism. Anal Bioanal Chem 416:1517–1525. https://doi.org/10.1007/s00216-024-05156-5

9. Zhang L, Wen S, Khan JU, et al (2024) Ultrasensitive Rapid Antigen Test by Geometric Lateral Flow Assays and Highly Doped Upconversion Nanoparticles. Anal Chem 96:16581–16589. https://doi.org/10.1021/acs.analchem.4c02379

10. Hu G, Gao S, Han X, Yang L (2020) Comparison of Immunochromatographic Strips Using Colloidal Gold, Quantum Dots, and Upconversion Nanoparticles for Visual Detection of Norfloxacin in Milk Samples. Food Anal Methods 13:1069–1077. https://doi.org/10.1007/s12161-020-01725-3

11. Hu X, Liao J, Shan H, et al (2023) A novel carboxyl polymer-modified upconversion luminescent nanoprobe for detection of prostate-specific antigen in the clinical gray

zonebase by flow immunoassay strip. Methods 215:10–16. https://doi.org/10.1016/j.ymeth.2023.05.001

12. Yuan X, Tang Z, Liu F, Liu J (2026) A lateral flow immunoassay strip employing upconversion luminescence for quantitative detection of prostate-specific antigen. Microchimica Acta 193:105. https://doi.org/10.1007/s00604-025-07736-3

13. Shen M, Li N, Lu Y, et al (2020) An enhanced centrifugation-assisted lateral flow immunoassay for the point-of-care detection of protein biomarkers. Lab Chip 20:2626–2634. https://doi.org/10.1039/D0LC00518E

14. Kim H-M, Kim J, An J, et al (2021) Au–Ag assembled on silica nanoprobes for visual semiquantitative detection of prostate-specific antigen. J Nanobiotechnology 19:73. https://doi.org/10.1186/s12951-021-00817-4

15. Cai Y, Zhang S, Dong C, et al (2021) Lateral flow immunoassay based on gold magnetic nanoparticles for the protein quantitative detection: Prostate-specific antigen. Anal Biochem 627:114265. https://doi.org/10.1016/j.ab.2021.114265

16. Bock S, An J, Kim H, et al (2020) A Lateral Flow Immunoassay for Prostate-Specific Antigen Detection Using Silica-Coated CdSe@ZnS Quantum Dots. Bull Korean Chem Soc 41:989–993. https://doi.org/10.1002/bkcs.12099

17. Salminen T, Juntunen E, Talha SM, Pettersson K (2019) High-sensitivity lateral flow immunoassay with a fluorescent lanthanide nanoparticle label. J Immunol Methods 465:39–44. https://doi.org/10.1016/j.jim.2018.12.001
